# Long-Lived Organic Radicals Drive the Photodegradation of Plastic Additives in Microplastic-Derived Dissolved Organic Matter

**DOI:** 10.1021/acs.est.6c01544

**Published:** 2026-06-17

**Authors:** Yangjian Zhou, Yunyi Zeng, Japhet Cheuk-Fung Law, Yu Lei, Xin Yang, Kelvin Sze-Yin Leung

**Affiliations:** † Department of Chemistry, 26679 Hong Kong Baptist University, Kowloon Tong, Kowloon, Hong Kong 999077, China; ‡ School of Environmental Science and Engineering, Guangdong Provincial Key Laboratory of Environmental Pollution Control and Remediation Technology, 26469 Sun Yat-Sen University, Guangzhou 510275, China; § State Key Laboratory of Green Papermaking and Resource Recycling, State Environmental Protection Key Laboratory of Environmental Health Impact Assessment of Emerging Contaminants, School of Environmental Science and Engineering, 12474 Shanghai Jiao Tong University, Shanghai 200240, China; ∥ HKBU Institute for Research and Continuing Education, Shenzhen Virtual University Park, Shenzhen 518000, China

**Keywords:** microplastic, microplastic-derived dissolved organic matter, long-lived organic radicals, plastic additives, photolysis

## Abstract

Microplastic-derived dissolved organic matter (MP-DOM) plays an important role in aquatic environments; however, its influence on the photodegradation of plastic additives remains unclear. In this study, bisphenol A (BPA) was selected as a representative plastic additive to investigate the effects of polystyrene-derived DOM (PS-DOM) on the photodegradation kinetics and mechanisms of BPA at different concentrations. PS-DOM significantly enhanced the photodegradation of BPA, with the promoting effect becoming more pronounced at lower BPA concentrations. Kinetic modeling revealed that as BPA levels decrease from high to low, the dominant contributor to BPA photodegradation shifts from the excited triplet state of PS-DOM (generated *via* photosensitization) to long-lived organic radicals (LLORs). This finding highlights the indispensable role of LLORs in the photodegradation of trace-level BPA (nM−μM) in surface waters. Probe experiments revealed that the one-electron reduction potentials of LLORs generated by various MP-DOM are around 1.50 V, indicating that LLORs have the potential to degrade plastic additives with oxidation potentials below this range. Furthermore, correlation analysis revealed that variations in unsaturated functional groups and electron-donating moieties (e.g., tannins and lignins) within MP-DOM were key factors controlling the quantum yield coefficients of LLORs across different MP-DOM types. This study provides novel insights into the photochemical reactivity of MP-DOM and its potential role in regulating the environmental transformation of plastic additives.

## Graphical abstract

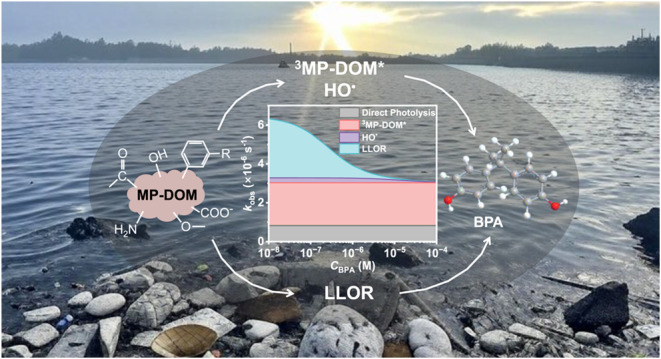

## Introduction

Plastic pollution has become a global environmental concern, with microplastics (MPs) receiving increasing attention due to their persistence, ubiquity, ecological risks, and potential to cause damage to human organs.^1,2^ During environmental aging processes, such as exposure to sunlight, thermal radiation, biodegradation, temperature fluctuations, and physical abrasion, MPs can release substantial amounts of microplastic-derived dissolved organic matter (MP-DOM).^3,4^ MP-DOM is an important anthropogenic source of global DOM. Its concentration can constitute up to 10% of the dissolved organic carbon (DOC) in surface seawater, and it exhibits toxicity that poses potential risks to aquatic ecosystems.^4^ As plastic production and usage continue to rise coupled with weak controls on plastic recycling, the proportion of MP-DOM in waterborne DOC is expected to increase further.^5,6^

Plastic additives, including plasticizers, stabilizers, and antioxidants, incorporated into polymers to enhance flexibility, durability, or stability, are recognized as the most hazardous components of MP-DOM.^7,8^ Furthermore, they account for more than 25% of its total mass.^7,8^ These additives, leach into surrounding environments during the aging and fragmentation of plastics.^8,9^ For example, bisphenol A (BPA) is an additive that is incorporated into materials such as polyvinyl chloride (PVC), polyethylene terephthalate (PET), low-density polyethylene (LDPE), polystyrene (PS), and polycarbonate (PC) to enhance strength, heat resistance, and corrosion resistance.^10–12^ The global release rate of BPA from marine plastics is estimated as 6.1 × 10^5^ kg yr^–1^.^13^ The released additives are often highly mobile and persistent, leading to their widespread occurrence in surface waters, where they pose significant toxicological risks because most organisms, including humans, are most active in the surface waters.^8,14^ For example, prolonged exposure to bisphenols and phthalate ester additives can disrupt hormone signaling and impair reproductive functions in aquatic organisms and humans.^14^ Given their diverse toxic effects, understanding the transformation behavior of plastic additives in natural systems, particularly within MP-DOM, is crucial for assessing their environmental fates and associated health risks.

Photodegradation is one of the primary degradation pathways for plastic additives in natural aqueous environments.^15^ Some plastic additives undergo direct photodegradation due to their intrinsic light absorption, whereas plastic additives lacking chromophoric structures are indirectly photodegraded through short-lived photooxidants (SLPOs, < 10 μs), such as singlet oxygen (^1^O_2_, 3.6 μs),^16^ hydroxyl radical (HO^•^, < 1.0 μs),^17^ and the excited triplet state of DOM (^3^DOM*, 1.6–6.3 μs),^18^ which are generated through photosensitization processes involving DOM.^19–21^ Within MP-DOM, functional groups including carbonyls, quinones, and aromatic moieties can absorb sunlight and generate ^3^DOM* of MP (^3^MP-DOM*), which subsequently transfer energy to dissolved oxygen to produce ^1^O_2_. HO^•^ are generated *via* Fenton-like reactions involving DOM or through the photolysis of polyhydroxylated aromatic compounds.^21–23^ In addition, recent studies have revealed that long-lived organic radicals (LLORs, i.e., > 10 μs), generated through the oxidation of electron-donating moieties in naturally derived DOM and in dissolved black carbon, can also participate in contaminant degradation.^18,24–28^ Unlike SLPOs, LLORs possess higher stability in water and can react over extended time scales, allowing them to significantly influence the fate of trace contaminants, particularly at environmentally relevant concentrations (<5.0 μM).^18,24–26,29^ MP-DOM contains abundant electron-donating moieties such as phenolic and aromatic amine structures, which have significant potential for LLOR formation.^30^ However, the mechanisms governing LLOR generation from MP-DOM under solar irradiation remain unclear, leading to limited understanding of the role of LLORs in the photochemical transformation of plastic additives within MP-DOM systems. This fundamental knowledge gap critically impedes accurate assessment of the environmental fate and risks of plastic additives in the presence of MP-DOM.

In this study, we systematically investigated the photodegradation kinetics and mechanisms of BPA at different concentrations, induced by photogenerated LLORs from PS-derived DOM (PS-DOM). A series of probes with varying oxidation potentials were employed to estimate the reduction potential of LLORs photochemically generated from different MP-DOMs, thereby elucidating the feasibility of LLOR-mediated degradation across diverse plastic additives. In addition, correlation analysis was used to identify the sources and mechanisms responsible for LLOR formation from various MP-DOMs. Overall, this study provides a comprehensive assessment of the photochemical generation of LLORs from MP-DOM, advancing our understanding of plastic additive photodegradation in the presence of MP-DOM.

## Materials and Methods

### Chemicals

PS, LDPE, polylactic acid (PLA), and poly­(butylene adipate terephthalate) (PBAT) were obtained from a plastic manufacturing company in Dongguan, China, with each MP having a particle size of less than 250 μm. Prior to use, the pristine MP particles were thoroughly washed with methanol and ultrapure water to minimize surface impurities. BPA (>99%) was purchased from Sigma-Aldrich. Ultrapure water (18.2 MΩ cm) was produced by a Millipore Milli-Q system and used for all experiments. Details of other chemicals used are summarized in Supporting Information Text S1.

### Preparation of MP-DOM

A concentration of 1.0 g L^–1^ of each of the four MPs was exposed to UVA radiation (UV intensity of 11.4 mW cm^–2^) for 18 h of photoaging. This treatment is approximately equivalent to 3–4 days of natural sunlight exposure in Hong Kong during summer (4.7 mW cm^–2^). After photoaging, the suspensions were filtered using a 0.45 μm glass fiber membrane. The filtered solutions were then subjected to solid-phase extraction (SPE) using a Bond Elut PPL cartridge (Agilent), yielding MP-DOM derived from the different types of MPs. The SPE method is described in Supporting Information Text S2.

### Experimental Procedures

The photochemical experiments of BPA were conducted in a solar simulator with 1000 W xenon lamp. The lamp was equipped with a 290 nm cutoff filter to produce simulated natural sunlight. Figure S1 shows the emission spectrum of the simulator compared with natural sunlight. The temperature was controlled at 28 ± 1 °C. Total photon irradiance was 3.23 × 10^–5^ Einstein cm^–2^ s^–1^ (Supporting Information Text S3). All MP-DOM test solutions (5.0 mg_C_ L^–1^) were placed in special quartz containers with 5.0 mM of phosphate buffered solution and buffered to a pH of 8.0 ± 0.1. The conditions enabled definitive identification of the underlying mechanisms. After addition of BPA (0.1–15 μM), the quartz was placed in the photolysis chamber and sampled at different time points. The collected samples were filtered and analyzed using high performance liquid chromatography (HPLC) or liquid chromatography–mass spectrometry (LC-MS). All experiments were performed in duplicate. The calculation of the light screening effect factor of MP-DOM can be found in Supporting Information Text S4.

### Analytical Methods

The DOC content of MP-DOM was quantified using a total organic carbon analyzer (Shimadzu TOC-V CPH). The ultraviolet–visible (UV–vis) absorption spectra and excitation–emission matrix (EEM) fluorescence spectra of MP-DOM were measured using a UV–vis spectrophotometer (Shimadzu UV-2700, Japan) and a fluorescence spectrophotometer (Shimadzu RF-5301, Japan), respectively. The molecular composition of MP-DOM was characterized by Fourier transform ion cyclotron resonance mass spectrometry (FT-ICR-MS, Bruker Solarix XR 7.0T, Germany). HO^•^ and ^1^O_2_ were detected by electron paramagnetic resonance (EPR, Bruker EMXplus spectrometer, Germany) using 5,5-dimethyl-1-pyrroline-*N*-oxide (DMPO) and 2,2,6,6-tetramethyl-4-piperidone (TEMP) as the respective trapping agents. A laser flash photolysis (LFP, LP980, Edinburgh Instruments, U.K.) system was employed to identify the generation of triplet states and LLORs in the PS-DOM system. The analysis details are reported in Supporting Information Text S5.

## Results and Discussion

### Photodegradation Kinetics of BPA

Using PS-DOM as a representative example, the photodegradation behavior of BPA at different concentrations (0.1–15 μM) in the presence of PS-DOM was investigated (Figure 1). In the absence of PS-DOM, BPA showed only slow and limited photodegradation over the entire concentration range (0.1–15 μM), with pseudo first-order rate constants of 5.47–6.15 × 10^–5^ min^–1^ (Figure S3). The slow photodegradation of BPA under simulated sunlight can be attributed to its weak absorption at wavelengths >290 nm (Figure S2). After the addition of 5.0 mg_C_ L^–1^ PS-DOM, the photodegradation rate constant (*k*_obs_, unit, min^–1^) increased markedly as the BPA concentration decreased (Figure 1c). In particular, when the BPA concentration was in the range of 5.0 – 15 μM, the *k*_obs_ at 5.0 μM was only approximately 1.17-fold higher than that at 15 μM. In contrast, in the lower concentration range of 0.1–5.0 μM, the *k*_obs_ at 0.1 μM was 1.88-fold higher than that at 5.0 μM. These results suggested that PS-DOM strongly promotes BPA photodegradation through indirect photodegradation and that this promotion is more pronounced at environmentally relevant low concentrations. The nonlinear relationship between BPA concentration and its *k*_obs_ leads to the postulation that the steady-state concentrations of photoproduced reactive intermediates (PPRIs) differ at different BPA concentrations.

**Figure 1:**
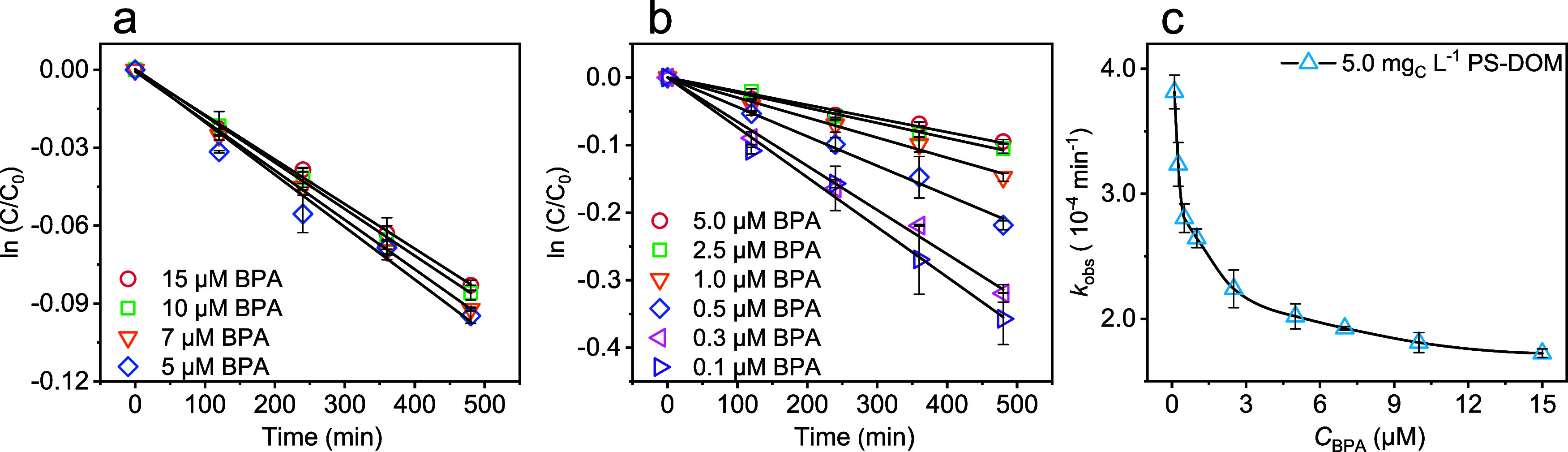
(a) Photodegradation kinetics of high concentration BPA (5.0–15 μM) in the presence of PS-DOM. (b) Photodegradation kinetics of low concentration BPA (0.1–5.0 μM) in the presence of PS-DOM. (c) *k*_obs_ at various BPA concentrations. Conditions: [PS-DOM]_0_ = 5.0 mg_C_ L^–1^, pH 8.0.

### Photodegradation Mechanisms of BPA in the Presence of PS-DOM

To elucidate the mechanisms of PS-DOM-enhanced BPA photodegradation at different concentrations, quenching experiments were performed. Sodium azide (NaN_3_) was used as a quencher for both ^1^O_2_ and HO^•^, while isopropanol (IPA) served as a specific quencher for HO^•^. Sorbic acid (SA) was employed as a quencher for ^3^PS-DOM*.^31,32^ At a BPA concentration of 5.0 μM, the addition of excess IPA (50 mM) and NaN_3_ (50 mM) inhibited the indirect photodegradation rate by 15.1% and 16.0%, respectively (Figure 2a). When excess SA was added, the indirect photodegradation rate decreased by 92.1% (*k*_obs_ = 6.75 × 10^–5^ min^–1^), approaching that of direct photolysis of BPA (*k*_obs_ = 5.47 × 10^–5^ min^–1^). This observation indicated that at high BPA concentrations, ^3^PS-DOM* was the predominant PPRI responsible for PS-DOM-enhanced BPA photodegradation, followed by HO^•^, whereas the contribution of ^1^O_2_ was negligible. At a BPA concentration of 0.1 μM, the addition of IPA and NaN_3_ reduced the indirect photodegradation rate by 10.6% and 13.8%, respectively, whereas the addition of SA led to a 60.4% decrease (Figure 2b). The results suggested that, in addition to ^3^PS-DOM* and HO^•^ as contributors to BPA degradation at low concentrations, other potential PPRIs (e.g., LLORs) may contribute to the accelerated photodegradation of BPA at low concentrations.

**Figure 2:**
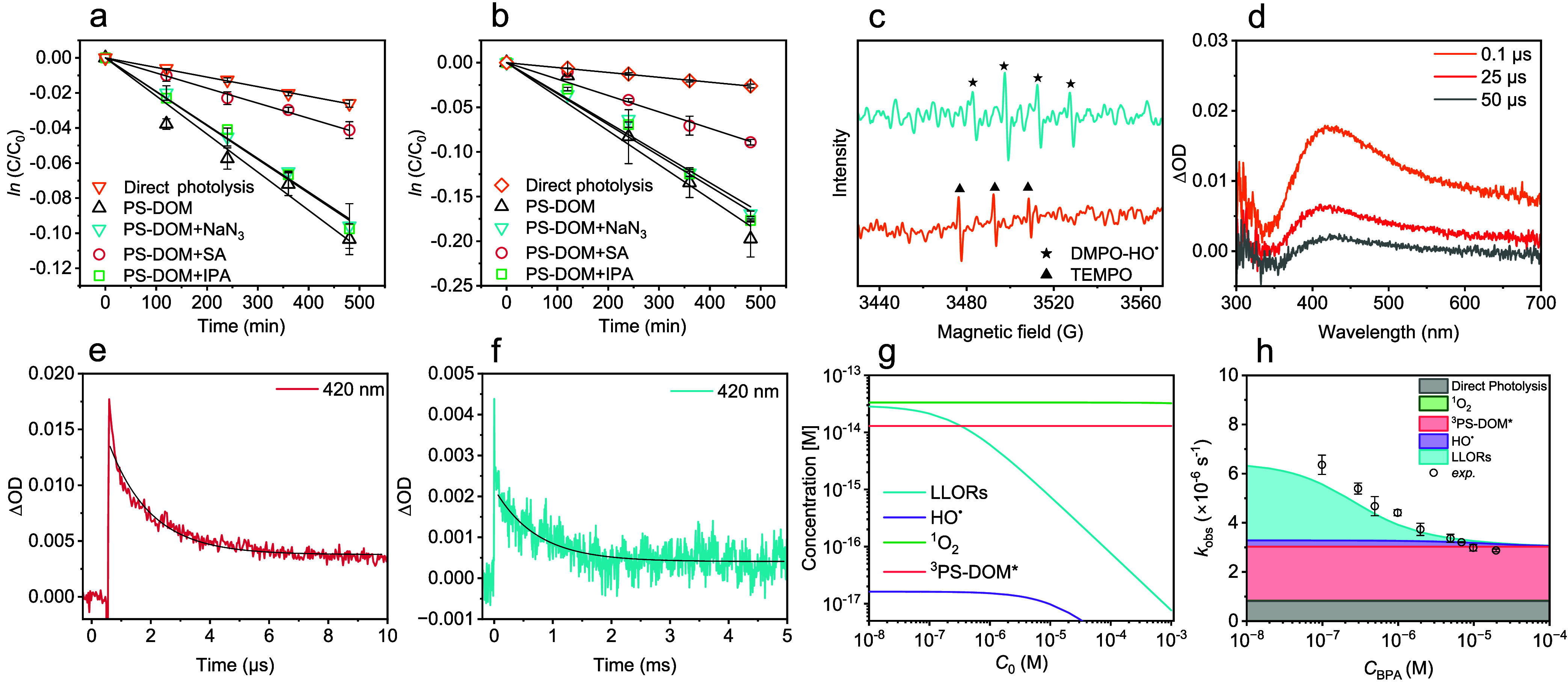
(a) Effects of different quenchers on the photodegradation kinetics of 5.0 μM BPA. (b) Effects of different quenchers on the photodegradation kinetics of 0.1 μM BPA. (c) EPR spectra of DMPO-HO^•^ and TEMPO obtained from PS-DOM system. (d) Transient absorption spectra obtained after 355 nm laser irradiation in the presence of PS-DOM. (e) Decay kinetics of ^3^PS-DOM* at 420 nm under time range of 0–25 μs. (f) Decay kinetics of LLORs at 420 nm under time range of 0–5 ms. (g) Simulated concentration differences of different PPRIs under various BPA concentrations. (h) Contributions of different PPRIs to BPA photodegradation at various BPA concentrations.

To verify the presence of these PPRIs, EPR and LFP analyses were performed. During the irradiation of PS-DOM, the addition of TEMP resulted in three EPR peaks with an intensity ratio of 1:1:1, corresponding to the TEMPO spin adduct. In addition, upon the addition of DMPO, four EPR peaks with an intensity ratio of 1:2:2:1 were observed, which are characteristic of the DMPO-HO^•^ adduct (Figure 2c). The combination indicated the generation of ^1^O_2_ and HO^•^ in PS-DOM under simulated sunlight irradiation. Furthermore, upon the addition 355 nm laser excitation of PS-DOM, a strong absorption peak at 420 nm appeared on the microsecond time scale and gradually decayed; it was assigned to the ^3^PS-DOM* (Figure 2d, e).^31^ However, fitting of the decay curve revealed that the signal did not fully follow pseudo first-order kinetics, implying that, in addition to ^3^PS-DOM*, other PPRIs were involved. When the observation time scale was extended, a signal with a lifetime of approximately 0.81 ms was detected, which was assigned to LLORs (Figure 2f).

To further validate the contributions of different PPRIs at varying BPA concentrations, kinetic modeling was applied. Previous studies reported that ^3^DOM* can oxidize contaminants, forming intermediates that are subsequently reduced back to the parent compound by DOM, thus inhibiting the degradation of the contaminant.^31,33^ To assess whether this process influences the current system, we utilized the model developed by Wenk et al.^34,35^ Our findings indicated that as the concentration of PS-DOM increased, there was no significant difference in the photodegradation rate of BPA (Figure S4), thus ruling out the possibility that PS-DOM reduces BPA intermediates. Assuming that at high BPA concentrations the photodegradation was governed solely by SLPOs, the total formation rate (*R*_SLPO_) and second-order rate constant between SLPOs and BPA (*k*_SLPO, BPA_) were calculated from eq 1 as 8.26 × 10^–10^ M s^–1^ and 2.18 × 10^9^ M^–1^ s^–1^, respectively (Figure S5). The *k*_SLPO, BPA_ is close to second-order rate constant of ^3^CBBP* and BPA (1.43 × 10^9^ M^–1^ s^–1^, Figure S16), highlighting the significance of ^3^PS-DOM* in the photodegradation of BPA by SLPOs. In contrast, at low BPA concentrations, where both SLPOs and LLORs were assumed to contribute to BPA degradation, the total formation rate of LLORs (*R*_LLOR_) and their second-order rate constant with BPA (*k*_LLOR, BPA_) were derived from eq 2 to be 8.19 × 10^–13^ M s^–1^ and 1.07 × 10^8^ M^–1^ s^–1^, respectively. The *k*_LLOR, BPA_ is close to that for reactions between phenoxy radicals and phenolic compounds (∼10^8^ M^–1^ s^–1^).^24^

1 $$\frac{1}{k_{\mathrm{obs},\mathrm{BPA}}^{\mathrm{SLPO}}}=\frac{1}{R_{\mathrm{SLPO}}}\left[\mathrm{BPA}\right]+\frac{k_{d}^{\mathrm{SLPO}}}{R_{\mathrm{SLPO}}k_{\mathrm{SLPO},\mathrm{BPA}}}$$

2 $$\frac{1}{k_{\mathrm{obs},\mathrm{BPA}}^{\mathrm{ind}}-k_{\mathrm{obs},\mathrm{BPA}}^{\mathrm{SLPO}}}=\frac{1}{R_{\mathrm{LLOR}}}\left[\mathrm{BPA}\right]+\frac{k_{d}^{\mathrm{LLOR}}}{R_{\mathrm{LLOR}}k_{\mathrm{LLOR},\mathrm{BPA}}}$$

Where *k*_d_^SLPO^ is the rate constant for the physical quenching of SLPO by water, *k*_obs, BPA_^SLPO^ and *k*_obs, BPA_^ind^ refer to the observed rate constants for the indirect photodegradation of BPA at high and low concentrations, respectively. [BPA] represents the concentration of BPA.

Based on the total formation rate of PPRIs divided by their consumption rate, the steady-state concentration of each PPRI ([PPRI]_SS_) was calculated (eq 3, Text S6). As shown in Figure 2g, with decreasing BPA concentration, the concentrations of LLORs and HO^•^ increased, indicating a BPA concentration-dependence of these species. In contrast, the concentrations of ^1^O_2_ and ^3^PS-DOM* were not influenced by the BPA concentration. This could be attributed to the predominant quenching of ^3^PS-DOM* by dissolved oxygen (*k* = 2.0 × 10^9^ M^–1^ s^–1^) and the low reactivity of ^1^O_2_ toward BPA (*k* = 3.0 × 10^5^ M^–1^ s^–1^).^36,37^

3 $${\left[\mathrm{PPRI}\right]}_{\mathrm{SS}}=\frac{R_{\mathrm{PPRI}}}{k_{d}^{\mathrm{PPRI}}+k_{\mathrm{PPRI},\mathrm{BPA}}\left[\mathrm{BPA}\right]}$$

4 $$k_{\mathrm{obs},\mathrm{PPRI}}=k_{\mathrm{PPRI},\mathrm{BPA}}{\left[\mathrm{PPRI}\right]}_{\mathrm{SS}}$$

5 kobs,BPA=kdir+kHO•+kO21+kPS−DOM*3+kLLOR=kdir+RHO•1kHO•,BPA(kdHO•+kPS−DOM,HO•[PS−DOM])+[BPA]+RO211kO21,BPA(kdΔ+kO21,PS−DOM[PS−DOM])+[BPA]+RPS−DOM*3(kdPS−DOM*3)/kPS−DOM*3,BPA+[BPA]+RLLORkdLLOR/kLLOR,BPA+[BPA]

Where *k*_d_^PPRI^ represents self-decay rate, *k*_PPRI, BPA_ represents the second-order reaction rate constant of BPA with PPRI. *k*_HO•,BPA_, kO21,BPA , *k*_^3^PS‑DOM*BPA_, and *k*_LLOR, BPA_ are the second-order reaction rate constants of BPA with HO^•^, ^1^O_2_, ^3^PS-DOM* and LLOR, respectively. *R*_HO•_, RO21 , and *R*_^3^PS‑DOM*_ are the formation rate of HO^•^, ^1^O_2_, and ^3^PS-DOM*, respectively.

Using eqs 4–5, the contributions of individual PPRIs to BPA photodegradation at different BPA concentrations were calculated. As shown in Figure 2h, with decreasing BPA concentrations, the contribution of ^1^O_2_ consistently remained below 1%. The contributions of ^3^PS-DOM* decreased from 72.6% at 100 μM BPA to 34.9% at 10 nM BPA. In contrast, the contribution of LLORs to BPA photodegradation increased from 0.3% at 100 μM BPA to approximately 48.1% at 10 nM BPA, which can be attributed to the BPA concentration-dependence of [LLOR]_SS_ (Figure 2h). Specifically, when the concentration of BPA increases, LLORs are quenched by BPA, resulting in a decrease in [LLOR]_SS_. Although HO^•^ also exhibited BPA concentration-dependence, its contribution to BPA photodegradation remained below 10%. When the predicted *k*_obs_ values were compared with the experimentally measured *k*_obs_ values, the error was found to be within 15%, confirming the reliability of the kinetic model and the contributions of various PPRIs in the BPA photodegradation process. In addition, the results predicted by the model were in good agreement with those obtained from the quencher experiments (Figure 2). Overall, we can conclude that LLORs play a dominant role in BPA photodegradation at low BPA concentrations (<1.0 μM), whereas ^3^PS-DOM* predominates at higher concentrations (>1.0 μM).

### Effect of LLORs of MP-DOM on the Photodegradation of Plastic Additives

Considering the low environmental concentrations of plastic additives (ng−μg L^–1^ levels), the important role of LLORs in BPA photodegradation cannot be neglected.^13^ To further elucidate the reaction mechanism between LLORs and plastic additives, the characteristics of LLORs generated from PS-DOM under simulated sunlight were investigated. Because LLORs are a complex mixture of various organic radicals derived from PS-DOM, a series of plastic additives and phenols including 2,4,6-trimethylphenol (TMP), 3,4-dimethoxyphenol (DMOP), phenol (PhOH), 4-methylphenol (4-MP), 4-hydroxybenzoic acid (4-CP) and trolox with different one-electron reduction potentials (*E*_red_^0^) were used as molecular probes to determine the oxidant capacity of LLORs generated from PS-DOM under simulated sunlight (Table S1). The enhancement factor (EF), defined as the ratio of *k*_obs_ at the low initial concentration (0.1 μM, *k*_obs, 0.1 μM_) to that at the high initial concentration (5.0 μM, *k*_obs, 5.0 μM_) of probes, was employed to examine the role of LLORs in PS-DOM solutions, as described in eq 6.^18,24,26^

6 $$\mathrm{EF}=\frac{k_{\mathrm{obs,}0.1\mathrm{\mu M}}}{k_{\mathrm{obs,}5.0\mathrm{\mu M}}}$$

An EF value greater than 1.0 means that LLORs can promote the photodegradation of plastic additives at low concentrations. An EF ≤ 1.0 means that LLORs show little or no contribution to degradation. This behavior can be attributed to low accumulated concentration of LLORs. Specifically, the limited availability of LLOR precursors restricts the concentration of photogenerated LLORs, allowing the degradation of only ∼nM levels of additives. At elevated additive concentrations, LLORs are insufficient to fully degrade additives; instead, most LLORs are quenched by additives, and the remaining additives subsequently reacts with SLPOs.^18,24,26^ As shown in Figures 3 and S6, except for 4-CP (EF = 1.0), all other tested probes exhibited EF values greater than 1.0 in the irradiated PS-DOM system. Among them, trolox showed the highest EF (4.24), followed by DMOP, TMP, 4-MP, and PhOH, indicating that LLORs exhibit selective reactivity toward different plastic additives. Considering the one-electron oxidation potentials (*E*_oxi_^0^) of 4-CP, PhOH, 4-MP, TMP, DMOP, and trolox are −1.56, −1.50, −1.38, −1.22, −1.17, and −0.95 V vs SHE, respectively,^18,26^ it can be inferred that the *E*_red_^0^ of LLORs generated from PS-DOM under irradiation is around 1.50 V, suggesting that LLORs possess strong oxidizing capability. To further validate the estimated *E*_red_^0^ of LLORs produced by PS-DOM irradiation, additional plastic additives and contaminants were employed (Figure S7). The known *E*_oxi_^0^ of aniline, 4-(dimethylamino) benzonitrile, and BPA are −1.02, −1.34 and −1.40 V vs SHE, respectively.^18^ Their corresponding EF values were 3.28, 1.83 and 1.88, respectively, supporting a conclusion that the reduction potential of LLORs derived from PS-DOM is around 1.50 V.

**Figure 3:**
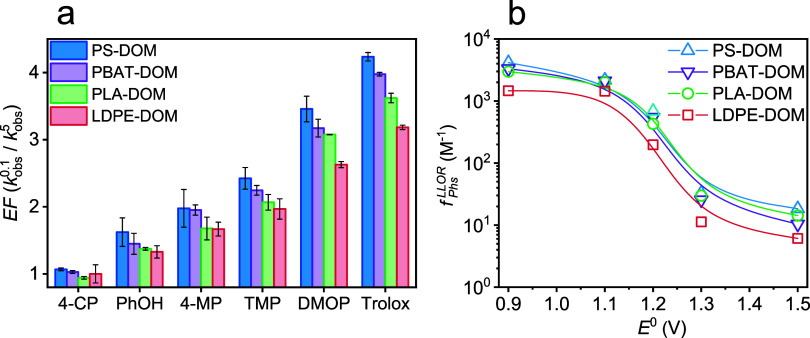
(a) Observed EF values of different probes in various MP-DOM systems. (b) Quantum yield coefficients of LLORs at different *E*_red_^0^ in various MP-DOM systems. Conditions: [MP-DOM]_0_ = 5.0 mg_C_ L^–1^, pH 8.0.

Subsequently, various probes were employed to investigate the LLORs generated from the other three MP-DOMs (PLA-DOM, PBAT-DOM, and LDPE-DOM) systems (Figures 3 and S6). Similar to the PS-DOM system, except for 4-CP, the photodegradation of all probes at 0.1 μM concentration was faster than that at 5.0 μM, and the EF values followed the same order: trolox > DMOP > TMP > 4-MP > PhOH. However, for a given probe, the EF values varied substantially among the different MP-DOM systems, implying that the physicochemical property of MP-DOM strongly affects LLOR reactivity. For instance, the EF value of DMOP reached 3.46 in the PS-DOM system, whereas it was only 2.63 in the LDPE-DOM system, indicating that PS-DOM-derived LLORs possess higher oxidative strength than those from LDPE-DOM.

To compare the differences in LLOR formation under simulated sunlight from various MP-DOM systems, the quantum yield coefficients of LLORs (*f*_Phs_^LLOR^) for each MP-DOM system were further calculated based on different probes. As shown in Figure 3b, they followed the same probe-dependent sequence as EF: trolox > DMOP > TMP > 4-MP > PhOH. In the PS-DOM irradiation system, the *f*_Phs_^LLOR^ of trolox was 231 times that of PhOH, reaffirming the selective reactivity of LLORs. Among the four MP-DOM systems, the *f*_Phs_^LLOR^ decreased in the order PS-DOM > PBAT-DOM > PLA-DOM > LDPE-DOM, revealing that aromatic MPs generate LLORs more efficiently than aliphatic MPs.

### Generation Mechanisms of LLORs in MP-DOM System Under Simulated Sunlight

#### Determination of MP-DOM Properties

Since LLORs originate from the photooxidation of various moieties within DOM, their generation efficiency and concentration are expected to strongly depend on the chemical composition and photochemical reactivity of the parent MP-DOM. To determine whether the greater LLORs yield observed with MP-DOM was associated with its inherent properties, the photophysical and photochemical properties and molecular signatures of MP-DOMs were compared. UV–vis spectra were used to determine E2/E3 and SUVA_254_, while EEM fluorescence spectroscopy provided the relative fluorescence areas of different regions. The electron-donating capacity (EDC) was used to characterize the reducing capacity of MP-DOMs (Text S7). Among the four MP-DOMs, LDPE-DOM exhibited the lowest E2/E3 and SUVA_254_ values (2.93 and 0.11 L mg_C_^–1^m^–1^, respectively, Figure S9), indicating its lower aromaticity and molecular weight, which can be attributed to the fully saturated structure of the LDPE polymer matrix. All four MP-DOMs showed strong fluorescence intensities in Regions III and V, suggesting a high content of fulvic- and humic-like substances (Figures S10,S11). Notably, PS-DOM exhibited the highest fluorescence intensity across all regions, followed by PBAT-DOM and PLA-DOM, whereas LDPE-DOM showed the weakest fluorescence. This trend was consistent with their corresponding EDC values, implying that fluorescence intensity and redox activity are positively correlated among MP-DOMs.^38^

In addition, FT-ICR-MS analysis was employed to obtain molecular parameters such as weighted oxygen-to-carbon ratio (O/C_w_), weighted hydrogen-to-carbon ratio (H/C_w_), weighted nominal oxidation state of carbon (NOSC_w_), weighted double bond equivalent (DBE_w_), weighted modified aromaticity index (AImod_w_), and weighted molecular weight (MW_w_) (Table S3). Both PS-DOM and PBAT-DOM showed relatively higher O/C_w_ (0.502 and 0.506), DBE_w_ (9.537 and 7.219), and AI_modw_ (0.338 and 0.156) values, and lower H/C_w_ (1.112 and 1.384) values compared with PLA-DOM and LDPE-DOM, whose corresponding O/C_w_, DBE_w_, AImod_w_, and H/C_w_ values were 0.490 and 0.437, 6.553 and 5.435, 0.114 and 0.147, and 1.467 and 1.475, respectively. These results indicated that PS-DOM and PBAT-DOM have components that are more aromatic, more unsaturated and more oxidized (such as ethers, carboxylic acids, and esters), whereas PLA-DOM and LDPE-DOM have lower levels of these components. These molecular patterns are consistent with the intrinsic chemical structures of the parent MPs. PS and PBAT contain aromatic components, whereas PLA and LDPE lack aromatic structures and unsaturation (Figure S8). However, because PLA contains unsaturated C=O bonds, this confers a higher degree of unsaturation in PLA-DOM compared with LDPE-DOM. The relative abundances of different compound classes, such as carbohydrates, aminosugar-like compounds, saturated compounds (Sat Com), tannin-like compounds, lignin-like compounds, unsaturated hydrocarbons (Unsat Hydrocar), and condensed aromatics (Con Aro), were also quantified (Figures S12,S13). Among them, PS-DOM and PBAT-DOM contained higher proportions of unsaturated tannin- and lignin-like compounds which are important components of fulvic- and humic-like substances, while PLA-DOM and LDPE-DOM exhibited relatively higher proportions of saturated compounds. Overall, the FT-ICR-MS findings show good consistency with the UV–vis and fluorescence spectra results.

The apparent quantum yields of SLPO (Φ_SLPO_) generation from the four MP-DOMs were determined using FFA, TA, and TMP as probes for ^1^O_2_, HO^•^, and ^3^MP-DOM*, respectively (Text S5, Figure S14). The Φ_^1^O_2__, Φ_HO•_, and *f*_TMP_ of four MP-DOMs were 2.8–9.5 × 10^–2^, 0.4–1.8 × 10^–5^, and 135.9–291.6 M^–1^, respectively. The Φ_^1^O_2__, Φ_HO•_, and *f*_TMP_ followed the order PS-DOM > PBAT-DOM > PLA-DOM > LDPE-DOM. This order confirmed that the formation of SLPOs is closely linked to the degree of unsaturation and oxidation level of MP-DOMs, with more oxidized and unsaturated DOM favoring higher SLPO generation.

#### The Correlation of the Generation LLORs and MP-DOM Properties

Correlation analyses were then performed between *f*_Phs_^LLOR^ and the measured photophysical, photochemical, and molecular characteristics of the MP-DOM samples. The apparent quantum yields of probes with different redox potentials reflect the relative levels of LLOR production with varying oxidation capacities. As shown in Figure 4, O/C_w_, tannin- and lignin-like intensity, EDC, fluorescence region V intensity, Φ_^1^O_2__, Φ_HO•_, and *f*_TMP_ were positively correlated with the *f*_Phs_^LLOR^ (*R* ^2^ > 0.4, *p* < 0.05), whereas H/C_w_ and fluorescence region I exhibited negative correlations (*R* ^2^ > 0.4, *p* < 0.05). In contrast, the correlations with the other parameters were weak and not statistically significant. These relationships indicated that the generation of LLORs is primarily governed by the unsaturated tannin- and lignin-like components and electron-donating moieties within the MP-DOM, rather than by its aliphatic characteristics. Such tannin- and lignin-like components, being aromatic and oxygen-containing compounds, derive from the phototransformation processes of photoinduced chain scission of MPs and additive leaching. Although fluorescence region I showed a notable negative correlation with *f*_Phs_^LLOR^ of five probes, its contribution was excluded due to its low proportion (<1%) of the total fluorescence intensity (Figure S11). A higher O/C_w_ and stronger tannin and lignin intensities imply greater MP-DOM oxidation and more unsaturated components, such as humic- or tannin-like chromophores. Those components are more prone to form ^3^MP-DOM* upon irradiation and to participate in subsequent electron-transfer reactions. The enhancement of *f*_TMP_ further supports the intrinsic connection between ^3^MP-DOM* formation and LLOR generation.

**Figure 4:**
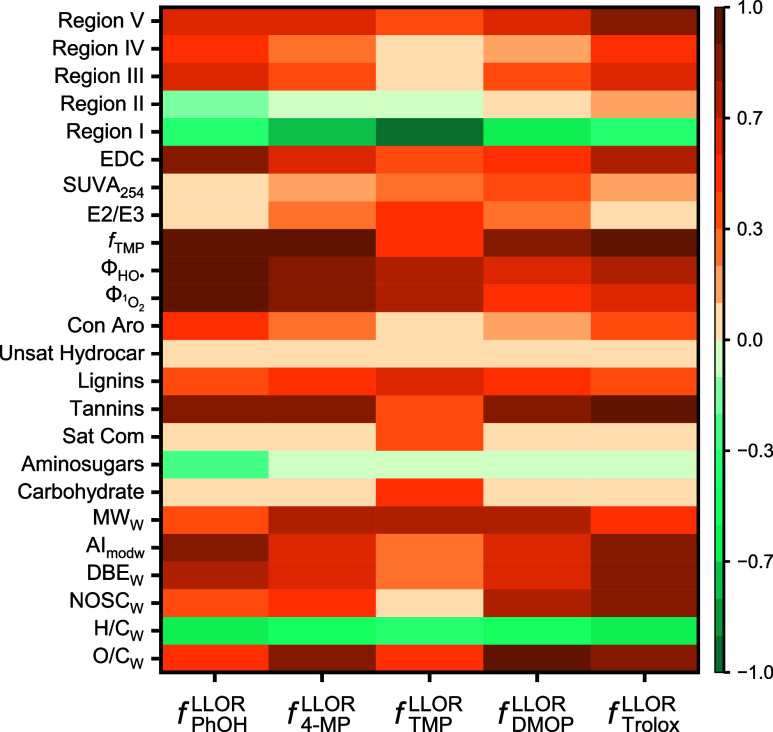
Correlation of the *f*_Phs_^LLOR^ and properties of various MP-DOM.

Although Φ_^1^O_2__ and Φ_HO•_ were positively correlated with *f*_Phs_^LLOR^, the addition of ^1^O_2_ and HO^•^ quenchers had negligible effects on BPA degradation (Figure 2). This finding suggests that ^1^O_2_ and HO^•^ are not the dominant reactive species in LLOR generation but rather covarying indicators associated with more unsaturated and more oxidized DOM. Previous studies have reported that ^3^DOM* can interact with electron-donating moieties of DOM via intramolecular electron transfer to form LLORs, such as semiquinone or phenoxyl radicals.^22,24,25^ The significant positive correlation between EDC and *f*_Phs_^LLOR^ observed in this study reinforces this mechanism. Therefore, it is reasonable to infer that the unsaturated chromophoric components of MP-DOM absorb photons to produce ^3^MP-DOM*, which subsequently undergoes intramolecular electron transfer to the electron-donating moieties, leading to the formation of LLORs dominated by one-electron oxidation processes. This mechanism accounts for the potential-dependent probe responses and highlights the critical role of aromatic or unsaturated, electron-rich MP-DOM substructures in driving LLOR generation.

## Environmental Impacts

Plastic additives represent one of the key toxicological concerns associated with MPs. Our findings reveal that MP-DOM plays a critical role in accelerating the photodegradation of plastic additives coreleased with it when exposed to simulated sunlight. The observed enhancement in photodegradation, which is most evident at low plastic additive concentrations (∼nM), highlights the significant role of LLORs (approximately 20–50%). This finding suggests that the environmental lifetime of trace-level (∼nM) plastic additives, such as BPA and PhOH, under natural sunlight exposure has likely been overestimated in previous assessments. Furthermore, given that the *E*_red_^0^ of LLORs derived from MP-DOM is around 1.50 V, the photolysis fates of plastic additives and contaminants with *E*_oxi_^0^ below this range should be re-evaluated, as their photochemical reactivity in natural environments may be greater than previously assumed. While our laboratory experiments suggest efficient interactions between MP-DOM and leached additives, environmental transport processes (e.g., advection, diffusion, and dilution) may spatially decouple these components in natural aquatic environments. Such separation could reduce reaction rates compared to those observed in confined laboratory systems. This factor should be further considered in future studies.

Correlation analysis confirmed that LLORs generated by MP-DOM irradiation primarily originate from its tannin- and lignin-like components. Since tannins and lignins are key components of plant structures such as trunks and leaves, these constituents can photochemically cleave to generate multiple organic radicals.^39,40^ These organic radicals, including phenoxy or semiquinone radicals, can degrade various environmental contaminants.^24,41–43^ Incorporating nontoxic, tannin- or lignin-like natural compounds into plastic formulations in the future could enhance LLOR generation and further promote the sunlight-driven degradation of plastic additives. This strategy offers a promising approach to reducing the persistence and ecological risks of plastic-associated contaminants in aquatic environments.

## Supplementary Material



## Notes

Details of chemicals; the calculation methods of photon irradiance; light screening effect factor; quantum yield and steady-state of PPRIs and MP-DOM’s electron-donating capacities; the photodegradation kinetics of BPA; and the property of MP-DOM (PDF)
