# Supplementary material for: Long-Lived Organic Radicals Drive the Photodegradation of Plastic Additives in Microplastic-Derived Dissolved Organic Matter
Source: Environ Sci Technol. 2026 Jun 17;60(26):18835–43. doi: 10.1021/acs.est.6c01544 (PMC13348169; doi:10.1021/acs.est.6c01544)
Supplement: Supplementary file 1 [file es6c01544_si_001.pdf]

Supporting Information

Long-Lived Organic Radicals Drive the Photodegradation of Plastic Additives in Microplastic-Derived Dissolved Organic Matter

Yangjian Zhou [a, b], Yunyi Zeng [a], Japhet Cheuk-Fung Law [a], Yu Lei [c], Xin Yang [b], Kelvin Sze-Yin Leung [a,d] *

[a] *Department of Chemistry, Hong Kong Baptist University, Kowloon Tong, Hong Kong SAR, China*

[b] *School of Environmental Science and Engineering, Guangdong Provincial Key Laboratory of Environmental Pollution Control and Remediation Technology, Sun Yat-sen University, Guangzhou 510275, China*

[c] *State Key Laboratory of Green Papermaking and Resource Recycling, State Environmental Protection Key Laboratory of Environmental Health Impact Assessment of Emerging Contaminants, School of Environmental Science and Engineering, Shanghai Jiao Tong University, Shanghai 200240, China*

[d] *HKBU Institute for Research and Continuing Education, Shenzhen Virtual University Park, Shenzhen 518000, China*

* Correspondence author: s9362284@hkbu.edu.hk (K. S-Y. Leung*)

This file contains 34 pages, including 7 texts, 3 tables, and 16 figures.

List of Contents

Texts:

Text S1. Chemicals

Text S2. MP-DOM extraction method

Text S3. Calculation of photon irradiance

Text S4. Determination of light screening effect factor of MP-DOM and indirect photodegradation rate constants

Text S5. Instrumental analysis for chemical quantification

Text S6. Quantification of photochemically produced reactive intermediates

Text S7. Quantifying the electron-donating capacities of the MP-DOM

Tables:

Table S1. Detection of plastic additives across different plastics

Table S2. HPLC methods for the analysis of individual compounds

Table S3. Intensity-weighted average values of the molecular composition of MP-DOM

Figures:

Figure S1. (a) Photodegradation kinetics of PNA (10 μM) in the presence of pyridine (10 mM) under simulated sunlight irradiation. (b) Irradiance spectrum of the solar simulator (290 − 600 nm) and the spectrum of natural sunlight in Hong Kong (114°17'E, 22°34'N, November, 2024, afternoon).

Figure S2. UV-vis absorbance spectra of MP-DOM and BPA at pH 8.0.

Figure S3. Photodegradation kinetics of BPA under simulated sunlight at pH 8.0.

Figure S4. Photodegradation of BPA in the presence of CBBP across different PS-DOM samples.

Figure S5. (a) Correlation between C_{BPA} and $1/k^{SLPO}_{obs,\ BPA}$. (b) Correlation between C_{BPA} and $1/k^{ind}_{obs,\ BPA}$ - $k^{SLPO}_{obs,\ BPA}$.

Figure S6. Photodegradation kinetics of various probes in different MP-DOM systems.

Figure S7. Photodegradation kinetics of aniline (a) and 4-(dimethylamino) benzonitrile (b) in

PS-DOM system.

Figure S8. Structure of the selected microplastics.

Figure S9. $SUVA_{254}$ (a), E2/E3 (b), and EDC (c) values of MP-DOM solution. $SUVA_{254}$ and E2/E3 obtained from the UV-vis spectra of the MP-DOM samples.

Figure S10. EEM of PS-DOM (a), PBAT-DOM (b), PLA-DOM (c), and LDPE-DOM (d).

Figure S11. Comparison of fluorescence intensities across different regions for PS-DOM (a), PBAT-DOM (b), PLA-DOM (c), and LDPE-DOM (d).

Figure S12. Van Krevelen diagrams depicting the molecular distribution and intensity characterization of PS-DOM (a), PBAT-DOM (b), PLA-DOM (c), and LDPE-DOM (d).

Figure S13. (a) Percentage intensity of different components in MP-DOM. (b) Percentage distribution of counts component in MP-DOM.

Figure S14. $\Phi_{^1O_2}$ (a), $\Phi_{HO\cdot}$ (b), and f_{TMP} values (c) of MP-DOM solution under simulated sunlight.

Figure S15. Photodegradation kinetics of various concentrations of TMP in PS-DOM (a), PBAT-DOM (b), PLA-DOM (c), and LDPE-DOM (d) irradiation systems. (e) Second-order kinetics of TMP with MP-DOM.

Figure S16. (a) Transient decay kinetics of $^3CBBP^*$ at 550 nm with different concentrations of BPA. (b) Plot of the first-order decay rate constant k_{obs} *vs.* BPA concentration.

Text S1 Chemicals

2,4,6-Trimethylphenol (TMP, 97%), 3,4-dimethoxyphenol (DMOP, 98%), 4-methylphenol (4-MP, >99%), phenol (PhOH, 99%), 4-hydroxybenzoic acid (4-CP, 99%), trolox (99%), terephthalic acid (TA, ≥99.0%), 2-hydroxyl terephthalic acid (2h-TA, ≥98.0%), 4-hydroxybenzophenone (CBBP, ≥98.0%), *p*-nitroanisole (PNA, ≥98.0%) and pyridine (≥99.0%) were obtained from Tokyo Chemical Industry Co., Ltd (Japan). Furfuryl alcohol (FFA, 98%), sorbic acid (SA, ≥99.0%), and 2′-azino-bis (3-ethylbenzthiazoline-6-sulfonic acid) (ABTS, >98%) were purchased from Sigma Aldrich (USA). Dipotassium hydrogen phosphate (Na_2HPO_4, 99%) and potassium dihydrogen phosphate (NaH_2PO_4, 99%) from Beijing Chemical Industry (China) was used to prepare phosphate buffer. Methanol (MeOH, LC-MS grade) and acetonitrile (ACN, LC-MS grade) were purchased from RCI Labscan (Thailand), while LC-MS grade formic acid (FA) (>99.0%) was purchased from Fisher Chemical (USA). Sodium hydroxide solution (NaOH) was obtained from Standard Chemical (Hong Kong SAR).

Text S2. MP-DOM extraction method

The filtered MP-DOM solution was acidified to pH 2.0 with formic acid. Two liters of the acidified sample were then passed through a solid-phase extraction (SPE) cartridge (Bond Elut PPL, 1 g, 6 mL; Agilent, USA) using a 16-position vacuum manifold set at a flow rate of 3.0 mL min^{-1}. After extraction, the cartridge was sequentially rinsed with 0.1% formic acid and ultrapure water, and then dried under vacuum. The retained organic matter was eluted from the cartridge with 10 mL of methanol and subsequently evaporated to dryness under a gentle stream of ultrapure nitrogen. The dried extract was collected and re-dissolved in ultrapure water prior to use.

Text S3. Calculation of photon irradiance

The irradiation intensity in the 290–600 nm ranges from a 1000 W xenon lamp (equipped with a 290 nm cutoff optical filter) was measured using the PNA–pyridine actinometer. The quartz cell containing pyridine (10 mM) and PNA (10 μM) with a total volume of 20 mL was placed in the sunlight simulator, and 1.0 mL samples were withdrawn at preset time points to measure PNA concentrations. The quantum yield of PNA (Φ_{PNA}) was calculated using eq. S1, and the total photon irradiance (unit, Einstein cm^{-2} s^{-1}) was calculated based on the pseudo first-order degradation rate constant of PNA ($k_{obs, PNA}$, Figure S1) according to eq. S2,

$$\Phi_{PNA} = 0.29[\text{pyridine}] + 0.00029 \quad (S1)$$

$$E^{0}_{p,tot} = \frac{k_{obs, PNA}[PNA]_0 l}{1000\Phi \sum \rho_\lambda (1-10^{-\varepsilon\lambda l [PNA]_0})\Delta\lambda} \quad (S2)$$

where $[PNA]_0$ is the initial PNA concentration (unit, M), ε_λ is the molar absorption coefficient of PNA at each wavelength (unit, $M^{-1}\cdot cm^{-1}$), l is the optical path length (unit, m), $\Delta\lambda$ is the wavelength resolution (unit, nm), and ρ_λ is the relative spectral photon irradiance.

The rate of light absorption (unit, Einstein L^{-1} s^{-1}) was calculated as follows:

$$R_a = \sum_{\lambda = 290\,nm}^{600\,nm} \frac{E^{0}_{P,\lambda}(1-10^{-Al})}{l} \quad (S3)$$

where A is the absorbance of the MP-DOM solution, $E^{0}_{P,\lambda}$ is the spectra photon irradiance at specific wavelength (unit, Einstein cm^{-2} s^{-1} nm^{-1}).

Text S4. Determination of light screening effect factor of MP-DOM and indirect photodegradation rate constants

To obtain actual direct photodegradation rate constants ($S_\lambda k_d$) in DOM, light screening effect of MP-DOM to direct photolysis was corrected by calculating light screening effect factor of MP-DOM (S_λ), as the UV-visible absorption spectra of the MP-DOM overlapped with that of BPA at $\lambda > 290$ nm (Figure S2). This result indicates that the MP-DOM would inhibit the direct photolysis of BPA through light screening effect.

Based on the UV-visible absorbance, S_λ was calculated with the following equations (S4 – S6):

$$S_\lambda = \frac{1-(10^{-(\alpha_\lambda+\varepsilon_\lambda[\mathrm{BPA}])l})}{2.303(\alpha_\lambda+\varepsilon_\lambda[\mathrm{BPA}])l} \quad (S4)$$

$$\Sigma S_\lambda = \frac{\Sigma E_\lambda \; S_\lambda \, \varepsilon_\lambda}{\Sigma E_\lambda \; \varepsilon_\lambda} \quad (S5)$$

$$k_{\mathrm{ind}} = k_{\mathrm{obs}} - S_\lambda \, k_{\mathrm{dir}} \quad (S6)$$

where α_λ is the light attenuation coefficient of MP-DOM (unit, m^{-1}), ε_λ is the molar absorption coefficient of BPA (unit, m^{-1} M^{-1}); [BPA] is the concentration of BPA (unit, μM); l is light path length (unit, m); E_λ is the light intensity (unit, Einstein m^{-2} s^{-1} nm^{-1}); k_{dir} is direct photolysis rate constant of BPA without MP-DOM at pH 8.0; k_{ind} is the indirect photodegradation rate constants of BPA caused by MP-DOM.

When the [BPA] decreased from 15 to 0.1 μM, the value of S_λ increased slightly from 0.96 to 0.97, indicating that the effect of [BPA] on S_λ is negligible.

Text S5. Instrumental analysis for chemical quantification

High performance liquid chromatography (HPLC)

The concentrations of probe compounds were analyzed using HPLC (Waters 2695, Waters, USA) equipped with photo-diode array and fluorescence detector and a ZORBAX HPLC ODS column (4.6 × 150 mm, 5 μm, Agilent, USA). The details are presented in Table S2.

Ultra high performance liquid chromatography-tandem mass spectrometry (UHPLC-QQQ-MS)

The concentration of BPA was analyzed using UHPLC-QQQ-MS with an Agilent 1290 UHPLC system coupled to an Agilent 6460 Triple Quadrupole mass spectrometer (Agilent Technologies, USA). The analysis was conducted on an ACQUITY BEH C18 column (100 × 2.1 mm, 1.7 μm, Waters, Ireland), with an injection volume of 5 μL. The electrospray ionization (ESI) interface was operated in negative ionization mode with a capillary voltage of 5.0 kV. The mobile phase consisted of ultrapure water and ACN, each containing 0.1% FA. The flow rate was set to 0.3 mL min^{-1}. The QQQ-MS was run under the following conditions: dry nitrogen gas at a flow rate of 10 L min^{-1} and a temperature of 280 °C; sheath gas at a temperature of 300 °C and a flow rate of 10 L min^{-1}; and a nebulizer pressure set to 45 psig. The precursor ion of BPA was m/z 227.1, with product ions at m/z 212.0 and 133.0. The corresponding collision energies (CE) were set at 12 and 20 V, with a dwell time of 200 ms and a fragmentor voltage of 45 V.

Fourier transform ion cyclotron resonance mass spectrometry (FT-ICR-MS)

Molecular-level information regarding the composition of MP-DOM was obtained using an FT-ICR-MS instrument equipped with a 7.0 T superconducting magnet system (SolariX XR, Bruker, Germany) and a quadrupole detector, operated in negative mode ESI. The MP-DOM sample was injected into the ESI source at a flow rate of 2 μL min^{-1}. The capillary voltage was set to 4.0 kV, with a -500 V end capillary voltage. The mass range was m/z 100 − 1200. Ions were accumulated in the collision chamber for 0.04 s, then transferred to the ICR cell with a flight time of 0.7 ms. A total of 300 scans with a 4M word size were performed to enhance the signal-to-noise ratio of the target peaks. To ensure the data quality of the FT-ICR-MS,

Suwannee River Natural Organic Matter was used as a standard for mass axis calibration prior to the sample analysis. During testing, the ion at m/z 311.168639 was used as an internal standard for real-time calibration.

Lase Flash Photolysis Technology

A laser flash photolysis system (LP980, Edinburgh Instruments, U.K.) equipped with a Nd: YAG laser (Quanta Ray, Spectra-Physics, U.S.) was used for radical kinetic determination and transient spectroscopy analysis. The 355 nm laser mode served as pump sources, and a 150 W xenon lamp provided the probe light. Samples were freshly prepared before each test. Transient signals were averaged over 12 – 16 laser shots, with duplicate tests performed for each sample.

Electron paramagnetic resonance

After simulated sunlight exposure, the sampled solution containing the trapping agents was transferred into a capillary tube (internal diameter ~0.55 mm), sealed with plasticine, and then placed into an EPR tube for measurement within the TM cavity. The EPR parameters were as follows: microwave frequency, 9.80 GHz (X-band); microwave power, 2.0 mW; power attenuation, 20.0 dB; modulation amplitude, 0.1 G at 100 kHz; time constant, 15.0 ms; and sweep width, 20 or 200 G. DMPO and TEMP were used as trapping agents for $HO^{\bullet}$ and $^1O_2$, respectively, and the corresponding mechanisms are shown in eqs. S7 – S8.

$$DMPO + HO^{\bullet} \rightarrow DMPO\text{-} HO^{\bullet} \quad (S7)$$

$$TEMP + {}^1O_2 \rightarrow TEMPO \quad (S8)$$

Fluorescence Spectra

The Excitation Emission Matrix (EEM) of MP-DOM was measured using an RF-5301 fluorescence spectrometer (Shimadzu, Japan). To obtain the EEM, the excitation wavelengths were incremented from 220 to 500 nm in 5-nm steps, while the emission was detected from 250 to 600 nm in 5-nm steps. The observed fluorescence peaks were categorized into distinct regions according to their excitation and emission wavelengths. Peaks at shorter excitation

wavelengths (< 250 nm) and shorter emission wavelengths (< 350 nm) are indicative of simple aromatic proteins like tyrosine (Regions I and II). Peaks at shorter excitation wavelengths (< 250 nm) but longer emission wavelengths (> 350 nm) correspond to fulvic acid-like substances (Region III). Peaks with intermediate excitation wavelengths (250−280 nm) and shorter emission wavelengths (< 380 nm) are associated with soluble microbial byproduct-like materials (Region IV). Lastly, peaks at longer excitation wavelengths (> 280 nm) and longer emission wavelengths (> 380 nm) are characteristic of humic acid-like organics (Region V).[1] This classification helps to identify and differentiate the types of organic materials present in the fluorescence spectra based on their spectral characteristics.

Text S6. Quantification of photochemically produced reactive intermediates

³MP-DOM*

Varied concentrations of TMP (50 to 250 μM) were employed as a probe molecule to investigate the electron transfer pathway in the ^{3}MP-DOM*-induced reaction (Figure S15). TMP follows pseudo-first-order kinetics with ^{3}MP-DOM* and shows limited reaction via direct photolysis with $^1O_2$ or HO•.[2, 3] The formation rate of ^{3}MP-DOM* ($R_{^3\text{MP-DOM*}}$, unit, M s^{-1}) can be obtained from eq. S9,

$$k_{\text{TMP}}=R_{^3\text{MP-DOM*}}\frac{k_{^3\text{MP-DOM*,TMP}}}{k_d^{^3\text{MP-DOM*}}+k_{^3\text{MP-DOM*,TMP}}[\text{TMP}]+k_{^3\text{MP-DOM*,O}_2}[\text{O}_2]} \quad (\text{S9})$$

where k_{TMP} is the observed degradation rate constant of TMP (unit, s^{-1}), $k_{^3\text{MP-DOM*,TMP}}$ is the second-order rate constant between ^{3}MP-DOM* and TMP (unit, M^{-1} s^{-1}), k_{O2} is the quenching rate constant for ^{3}MP-DOM* by dissolved oxygen (8.9 × 10^8 M^{-1} s^{-1}), $k_d^{^3\text{MP-DOM*}}$ is the physical quenching constant of ^{3}MP-DOM* (9.0 × 10^4 s^{-1}).[4, 5] Eq. S10 presents the linearized form of eq S9.

$$\frac{1}{k_{\text{TMP}}}=\frac{1}{R_{^3\text{MP-DOM*}}}[\text{TMP}]+\frac{k_d^{^3\text{MP-DOM*}}+k_{^3\text{MP-DOM*,O}_2}[\text{O}_2]}{R_{^3\text{MP-DOM*}}\,k_{^3\text{MP-DOM*, TMP}}} \quad (\text{S10})$$

The $R_{^3\text{MP-DOM*}}$ and $k_{^3\text{MP-DOM*,TMP}}$ values can be obtained from the slopes and intercepts of the linear plots of 1/k_{TMP} *versus* [TMP] (Figure S15).

The steady-state concentration of ^{3}MP-DOM* ([^{3}MP-DOM*]$_{ss}$, unit, M) was calculated in various BPA concentrations and 5.0 mgC L^{-1} MP-DOM following eq. S11.

$$[^3\text{MP-DOM*}]_{\text{SS}}=\frac{R_{^3\text{MP-DOM*}}}{k_d^{^3\text{MP-DOM*}}+k_{^3\text{MP-DOM*,BPA}}[\text{BPA}]+k_{^3\text{MP-DOM*,O}_2}[\text{O}_2]} \quad (\text{S11})$$

where $k_{^3\text{MP-DOM*,BPA}}$ is the second-order rate constant between ^{3}MP-DOM* and BPA (1.43 × 10^9 M^{-1} s^{-1}, Figure S16).

Because MP-DOM is a complex mixture of various cross-linked compounds, the ^{3}MP-DOM* formed under irradiation exhibit a wide range of properties and reactivities. The degradation rate of 10 μM TMP (used as a ^{3}MP-DOM* probe), divided by R_a, yields the quantum yield coefficient for electron transfer by ^{3}MP-DOM* (f_{TMP}, unit, M^{-1}), which can be calculated using eq. S12.

$$f_{\text{TMP}}=\frac{k_{\text{TMP}}}{R_a} \quad (\text{S12})$$

HO•

The formation rate of HO• ($R_{HO•}$, unit, M s^{-1}) was quantified by the addition of TA (10 μM, non-fluorescent) and the detection of 2h-TA (a fluorescent product), which is the single hydroxylation product of TA. This reaction has been shown to proceed with a yield of 35%.[6] Accordingly, $R_{HO•}$ and the steady-state concentration of HO• ($[HO^•]_{SS}$, unit, M) in the presence of TA were calculated using the following equation:

$$\frac{d[\text{2h-TA}]}{dt} = 0.35 R_{HO^•} \quad (S13)$$

The apparent quantum yield of HO• ($\Phi_{app,\ HO^•}$) was calculated as:

$$\Phi_{app,\ HO^•} = \frac{R_{HO^•}}{R_a} \quad (S14)$$

$[HO^•]_{SS}$ in the presence of BPA was calculated as:

$$[HO^•]_{SS} = \frac{R_{HO^•}}{k_{BPA,HO^•}[\text{BPA}] + k_{\text{MP-DOM},HO^•}[\text{MP-DOM}]} \quad (S15)$$

where $k_{BPA,\ HO^•}$ is the reaction rate constant between BPA and HO• (1.6×10^{10} M^{-1} s^{-1}).[7]

$^1O_2$

The formation rate of $^1O_2$ ($R_{^1O_2}$, unit, M s^{-1}) and the steady-state concentration of $^1O_2$ ($[^1O_2]_{SS}$ unit, M) were calculated following eqs. S16 – S17:

$$k_{FFA} = k_{FFA,\ ^1O_2}[^1O_2]_{SS} + k_{FFA,\ HO^•}[HO^•]_{SS} \quad (S16)$$

$$R_{^1O_2} = [^1O_2]_{SS}\, k_d^{\Delta} \quad (S17)$$

where k_{FFA} is the degradation rate of 10 μM FFA, k_d^{Δ} is the rate constant of physical quenching by water (2.76×10^5 s^{-1}), $k_{FFA,\ ^1O_2}$ is the reaction rate constant between FFA and $^1O_2$ (1.2×10^8 M^{-1} s^{-1}), $k_{FFA,\ HO^•}$ is the reaction rate constant between FFA and HO• (1.5×10^{10} M^{-1} s^{-1}).[8]

The apparent quantum yield of $^1O_2$ ($\Phi_{app,\ ^1O_2}$) was calculated as:

$$\Phi_{app,\ ^1O_2} = \frac{R_{^1O_2}}{R_a} \quad (S18)$$

$[^1O_2]_{SS}$ in the presence of BPA was calculated as:

$$[^1O_2]_{SS} = \frac{R_{^1O_2}}{k_{BPA,^1O_2}[\text{BPA}] + k_{^1O_2,\text{MP-DOM}}[\text{MP-DOM}] + k_d^{\Delta}} \quad (S19)$$

where $k_{BPA,\,^1O_2}$ is the reaction rate constant between BPA and $^1O_2$ (8.0×10^6 M^{-1} s^{-1}).[9]

LLORs

Due to the complexity of LLORs in illuminated MP-DOM, directly determining their photochemical quantum yield is challenging. Consequently, the quantum yield coefficient, denoted as f_{Phs}^{LLOR}, was applied in this study, similar to the quantum yield coefficient of triplets. Here, "Phs" refers to phenolic probes, including TMP, DMOP, PhOH, 4-MP, and trolox. f_{Phs}^{LLOR} is given by the following equation:[10]

$$f_{Phs}^{LLOR}=\frac{k_{0.1}^{obs,Phs}-k_{5.0}^{obs,Phs}}{R_a} \quad (S20)$$

where $k_{0.1}^{obs,Phs}$ and $k_{5.0}^{obs,Phs}$ are the observed degradation rate constant of the phenolic probes at 0.1 μM and 5.0 μM concentrations, respectively (unit, s^{-1}). The steady-state concentration of LLOR ($[LLOR]_{SS}$, unit, M) was calculated following eqs. S21:

$$[LLOR]_{SS} = \frac{R_{LLOR}}{k_d^{LLOR} + k_{LLOR,BPA}\,[BPA]} \quad (S21)$$

where $k_{LLOR,BPA}$ is the second-order reaction rate of LLOR and BPA (unit, M^{-1} s^{-1}), k_d^{LLOR} is the rate constant for physical quenching of LLOR by water (unit, s^{-1}), and R_{LLOR} represents the formation rate of LLOR (unit, M s^{-1}). At high concentrations of BPA (> 5.0 μM), degradation primarily occurs through SLPO (^{3}MP-DOM*, $HO^{\bullet}$ and $^1O_2$) generated by MP-DOM photosensitization. Consequently, the degradation of BPA at high concentrations was represented by eq. S22. The R_{SLPO} and $k_{obs,\,BPA}^{SLPO}$ values can be obtained from the slopes and intercepts of the linear plots of $1/k_{obs,\,BPA}$ *versus* [BPA]. At low concentrations of BPA, BPA is degraded not only by SLPO generated through MP-DOM photosensitization but also by LLOR. Therefore, the degradation of BPA at low concentrations is described by eq. S23.

$$\frac{1}{k_{obs,BPA}^{SLPO}} = \frac{1}{R_{SLPO}}[BPA] + \frac{k_d^{SLPO}}{R_{SLPO}\,k_{SLPO,BPA}} \quad (S22)$$

$$\frac{1}{k_{obs,BPA} - k_{obs,BPA}^{SLPO}} = \frac{1}{R_{LLOR}}[BPA] + \frac{k_d^{LLOR}}{R_{LLOR}\,k_{LLOR,BPA}} \quad (S23)$$

where R_{SLPO} represents the formation rate of SLPO (unit, M s^{-1}), and k_d^{SLPO} is the rate constant for the physical quenching of SLPO by water (unit, M^{-1} s^{-1}). The pseudo-first-order rate constants for SLPO and LLOR in the degradation of BPA are given by eq. S24.

$$k_{SLPO/LLOR} = k_{SLPO/LLOR,\,BPA}\ [SLPO/LLOR]_{SS} \quad (S24)$$

Thus, at different BPA concentrations, the relative contribution of SLPO and LLOR to the degradation of BPA is expressed by the following equation:

$$k_{\text{obs, BPA}} = k_{\text{dir}} + k_{\text{HO}^\bullet} + k_{^1\text{O}_2} + k_{^3\text{MP-DOM}^*} + k_{\text{LLOR}}$$

$$= k_{\text{dir}} + \frac{R_{\text{HO}^\bullet}}{(k_d^{\text{HO}^\bullet} + k_{\text{MP-DOM, HO}^\bullet}[\text{MP-DOM}])/k_{\text{HO}^\bullet,\text{BPA}} + [\text{BPA}]} + \frac{R_{^1\text{O}_2}}{(k_d^{\Delta} + k_{^1\text{O}_2,\text{MP-DOM}}[\text{MP-DOM}])/k_{^1\text{O}_2,\text{BPA}} + [\text{BPA}]}$$

$$+ \frac{R_{^3\text{MP-DOM}^*}}{(k_d^{^3\text{MP-DOM}^*})/k_{^3\text{MP-DOM}^*,\text{BPA}} + [\text{BPA}]} + \frac{R_{\text{LLOR}}}{k_d^{\text{LLOR}}/k_{\text{LLOR, BPA}} + [\text{BPA}]} \quad \text{(S25)}$$

where $k_{\text{HO}^\bullet,\text{BPA}}$, $k_{^1\text{O}_2,\text{ BPA}}$, and $k_{\text{LLOR, BPA}}$ are the second-order reaction rate constant of BPA with $\text{HO}^\bullet$, $^1\text{O}_2$ and LLOR, respectively (unit, $\text{M}^{-1}\ \text{s}^{-1}$).

Text S7. Quantifying the electron-donating capacities of the MP-DOM.

The electron-donating capacities (EDC) of MP-DOM were determined by quantifying the number of electrons transferred from the MP-DOM to the radical cation of ABTS ($ABTS^{\bullet+}$). $ABTS^{\bullet+}$ concentrations were quantified based on measured absorbances at 728 nm using the molar absorption coefficient ε (728 nm) = 14000 M^{-1} cm^{-1} .[11] To calculate the EDC, the residual $ABTS^{\bullet+}$ concentration in the reaction mixture containing MP-DOM was compared to that of a reaction mixture containing only the DOM-free blank and normalized to the DOC concentration of the sample in the reaction mixture (eq. S26)

$$EDC=\frac{A_{\mathrm{blank}} - A_{\mathrm{sample}}}{l\ \varepsilon_{\mathrm{ABTS}^{\bullet+}}\ c_{\mathrm{DOC}}} \quad \text{(S26)}$$

where A_{blank} and A_{sample} are the resulting absorbance values (λ = 728 nm) of the reaction mixtures containing the DOM-free blank and the DOM sample, respectively, $\varepsilon_{ABTS^{\bullet+}}$ is the molar absorption coefficient of $ABTS^{\bullet+}$ (unit, M^{-1} cm^{-1}), l is the optical pathlength (unit, cm), and c_{DOC} is the DOC concentration in the final reaction mixture (unit, $mg_C \cdot L^{-1}$).

Table S1. Detection of plastic additives across different plastics

	HDPE	LDPE	PE	PS	HIPS	PP	PS	PVC	PET	PA	PUR	Rubber	ABS	PC
BPA	√	√	√	√	NA	√	√	√	√	√	√	NA	√	√
PhOH	√	√	√	√	√	√	√	√	√	√	√	√	√	√
TMP	NA	NA	NA	NA	NA	NA	NA	NA	NA	NA	NA	NA	NA	NA
4-MP	√	NA	√	NA	NA	√	NA	NA	√	NA	NA	NA	NA	√
4-CP	√	NA	√	NA	NA	√	NA	NA	√	NA	NA	NA	NA	√
Aniline	NA	NA	√	√	NA	√	√	√	√	√	√	√	NA	√

Data source: https://doi.org/10.5281/zenodo.10701706

Table S2. HPLC methods for the analysis of individual compounds

Compound	ACN	Ultrapure water (0.1% FA)	Absorption wavelength (nm)	Excitation/Emission wavelength (nm)
TMP	80%	20%	220	230/325
DMOP	40%	60%	220	230/325
4-MP	80%	20%	220	225/316
PhOH	40%	60%	220	275/310
trolox	80%	20%	220	
FFA	40%	60%	220	-
2h-TA	40%	60%	-	250/410
PNA	40%	60%	313	-
aniline	70% (MeOH)	30% (Water)	-	232/343
DMABN	80%	20%	298	290/488
4-CP	60%	40%	210	275/310

Table S3. Intensity-weighted average values of the molecular composition of MP-DOM

MP-DOM	O/C_w	H/C_w	$NOSC_w$	DBE_w	$AImod_w$	MW_w
PS-DOM	0.502	1.112	-0.094	9.537	0.338	394.05
PBAT-DOM	0.506	1.384	-0.156	7.219	0.156	418.77
PLA-DOM	0.490	1.467	-0.486	6.553	0.114	425.33
LDPE-DOM	0.437	1.475	-0.591	5.435	0.147	336.68

Although PLA contains C=O bonds in its backbone, its lower O/Cw ratio compared with PS may be attributed to polymer-specific degradation pathways, as PLA photodegradation mainly generates low-molecular-weight carboxylic acids and oligomers that may not be fully represented in the FT-ICR-MS-assigned MP-DOM pool.

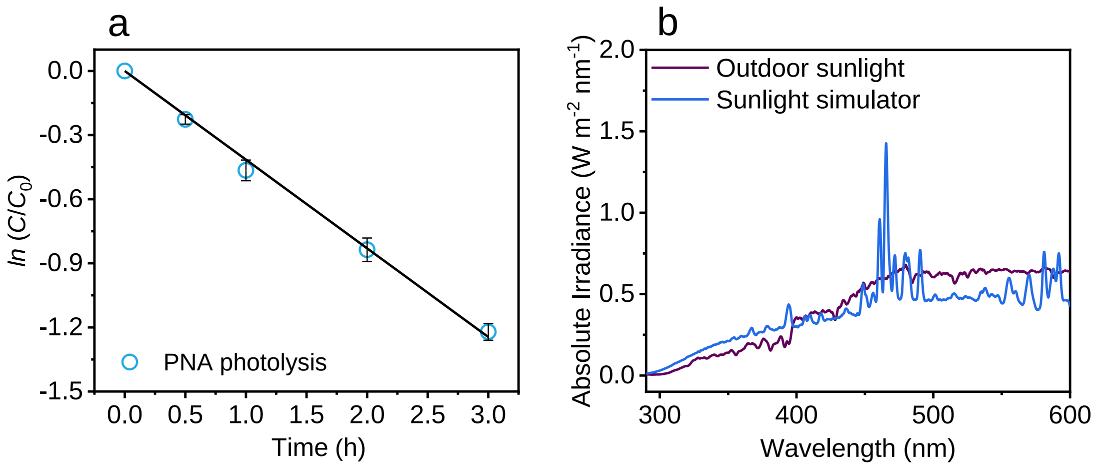

Figure S1. (a) Photodegradation kinetics of PNA (10 μM) in the presence of pyridine (10 mM) under simulated sunlight irradiation. (b) Irradiance spectrum of the solar simulator (290 – 600 nm) and the spectrum of natural sunlight in Hong Kong (114°17'E, 22°34'N, November, 2024, afternoon).

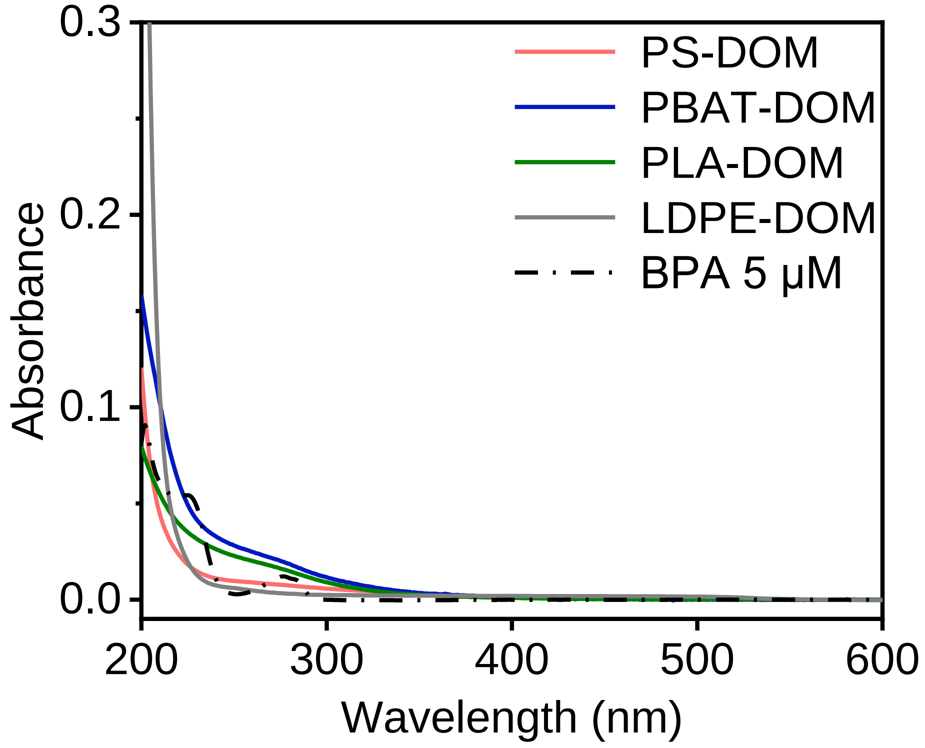

Figure S2. UV-vis absorbance spectra of MP-DOM and BPA at pH 8.0. Condition: $[\text{MP-DOM}]_0 = 5.0\ \text{mg}_\text{C}\ \text{L}^{-1}$.

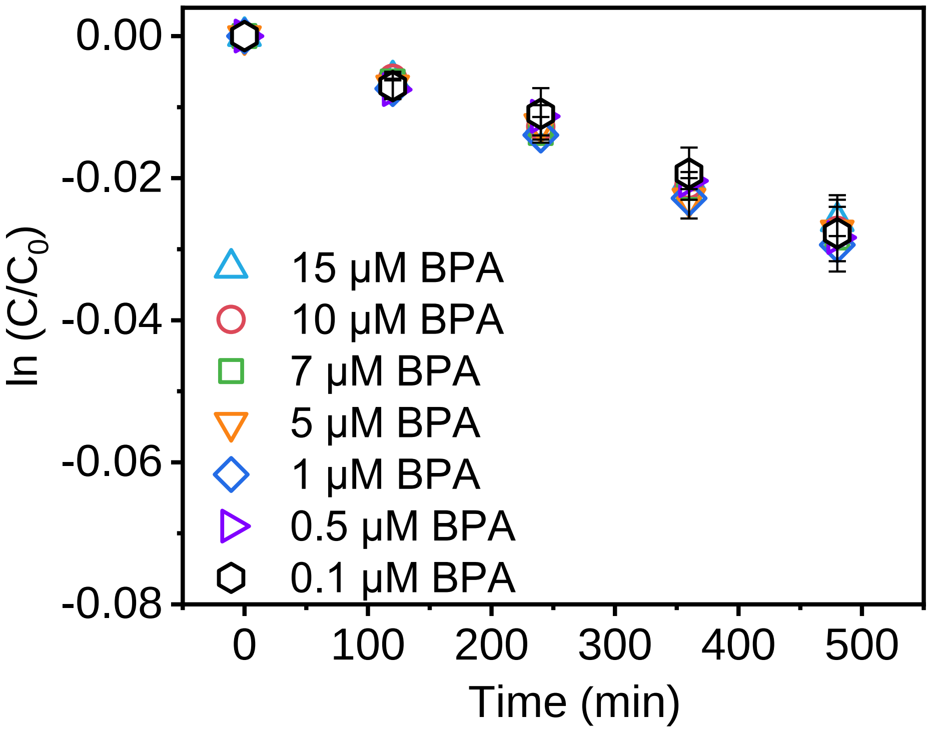

Figure S3. Photodegradation kinetics of BPA under simulated sunlight at pH 8.0.

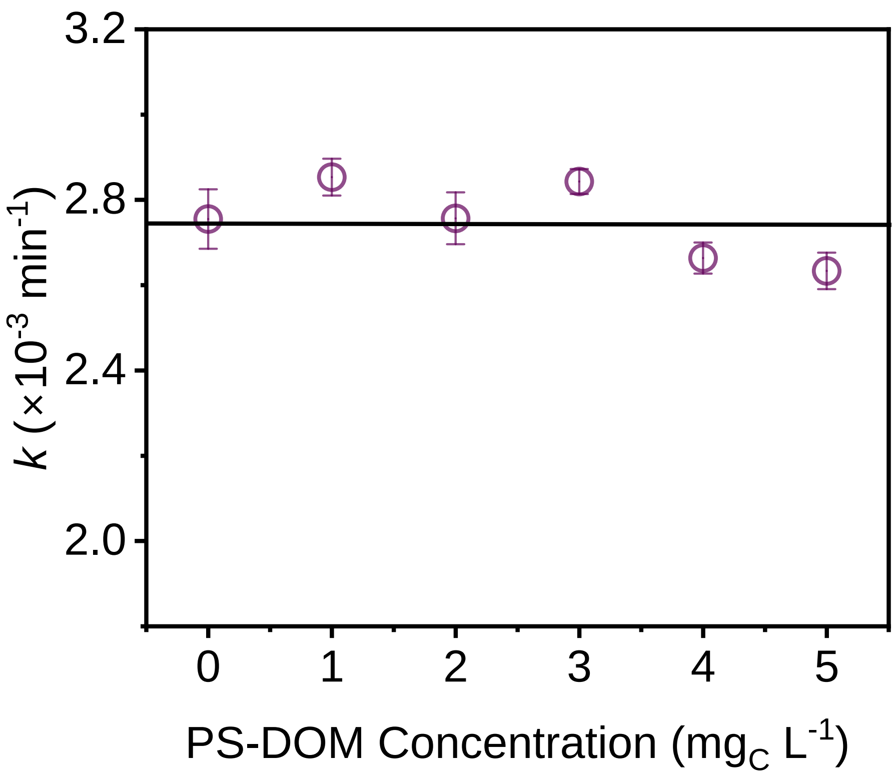

Figure S4. Photodegradation of BPA in presence of CBBP across different PS-DOM samples. Conditions: $[BPA]_0$ = 5.0 μM, $[CBBP]_0$ = 50 μM, pH 8.0.

Note: a gradual decrease in the apparent rate constant (*k*) indicates that PS-DOM is capable of reducing BPA intermediates. In contrast, the absence of a significant change in *k* suggests that PS-DOM is unlikely to participate in the reduction of BPA intermediates.

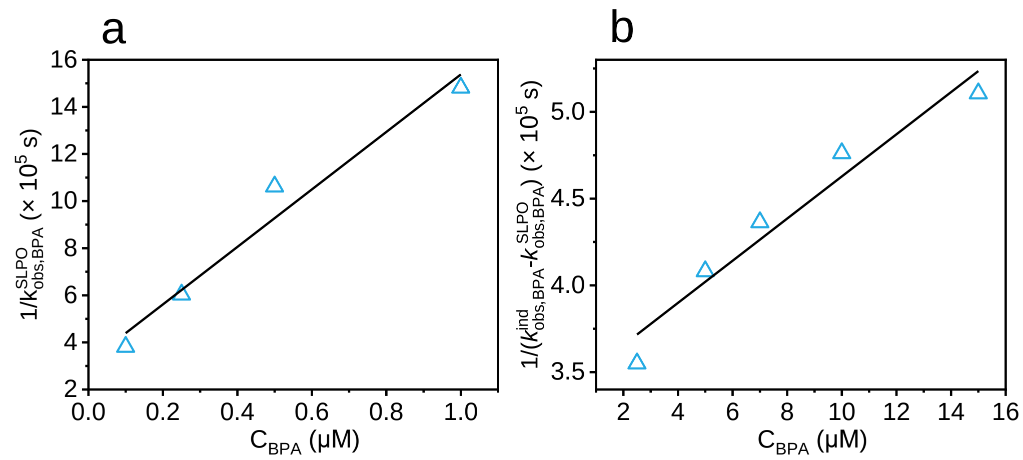

Figure S5. (a) Correlation between C_{BPA} and $1/k^{SLPO}_{obs,\ BPA}$. (b) Correlation between C_{BPA} and $1/k^{ind}_{obs,\ BPA}$ - $k^{SLPO}_{obs,\ BPA}$.

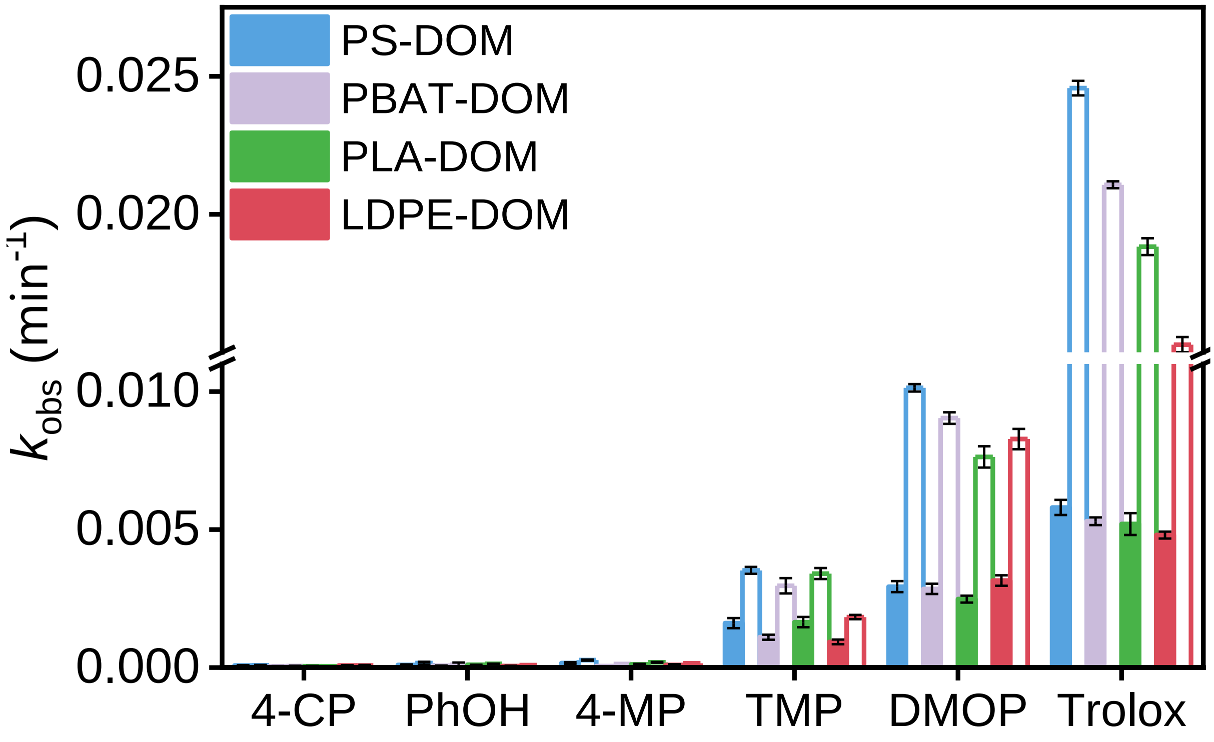

Figure S6. Photodegradation kinetics of various probes in different MP-DOM systems. Conditions: $[PS\text{-}DOM]_0$ = 5.0 mg_C L^{-1}, pH 8.0. Colored columns represent probe concentrations of 0.1 μM, while non-colored columns represent probe concentrations of 5.0 μM.

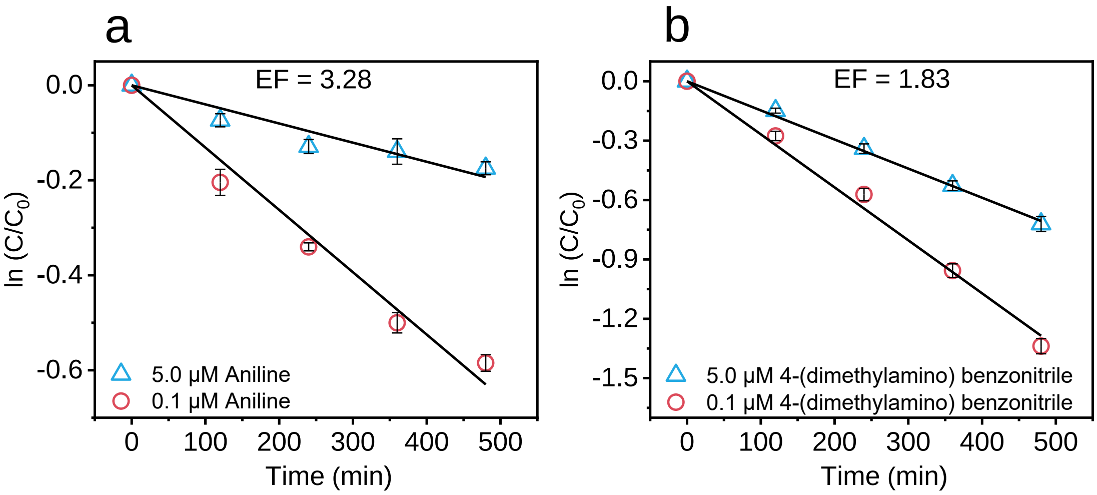

Figure S7. Photodegradation kinetics of aniline (a) and 4-(dimethylamino) benzonitrile (b) in PS-DOM system. Conditions: $[\text{PS-DOM}]_0 = 5.0\ \text{mg}_\text{C}\ \text{L}^{-1}$, pH 8.0.

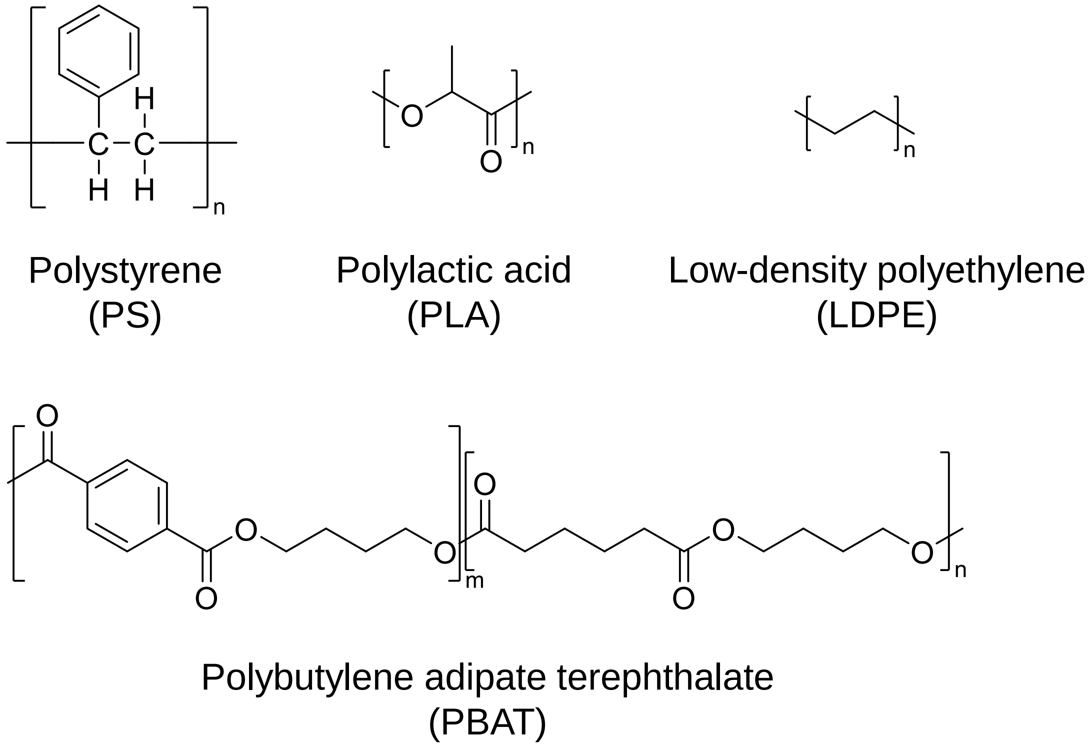

Figure S8. Structure of the selected microplastics.

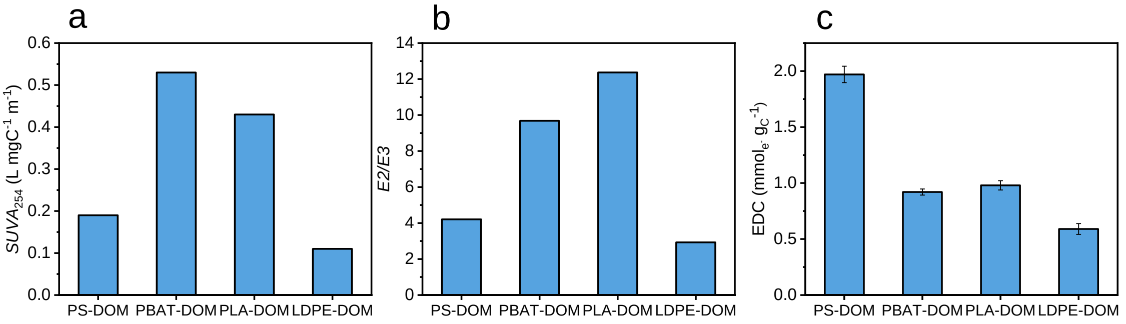

Figure S9. $SUVA_{254}$ (a), E2/E3 (b), and EDC (c) values of MP-DOM solution. $SUVA_{254}$ and E2/E3 obtained from the UV-vis spectra of the MP-DOM samples.

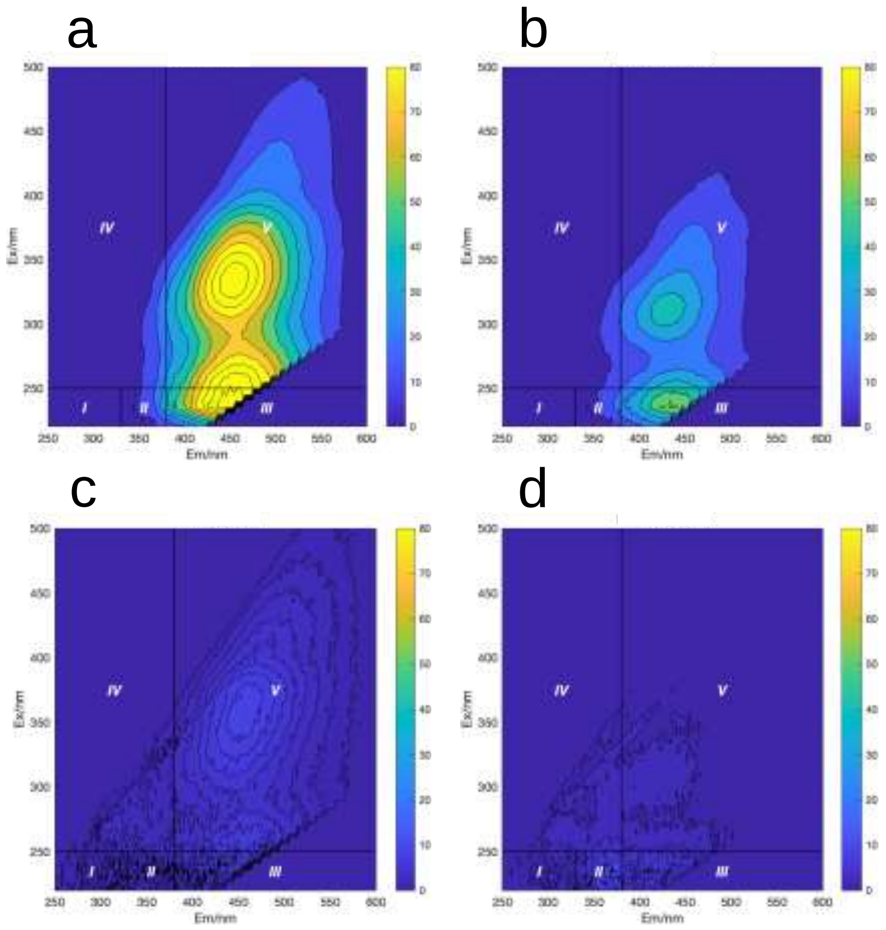

Figure S10. EEM of PS-DOM (a), PBAT-DOM (b), PLA-DOM (c), and LDPE-DOM (d).

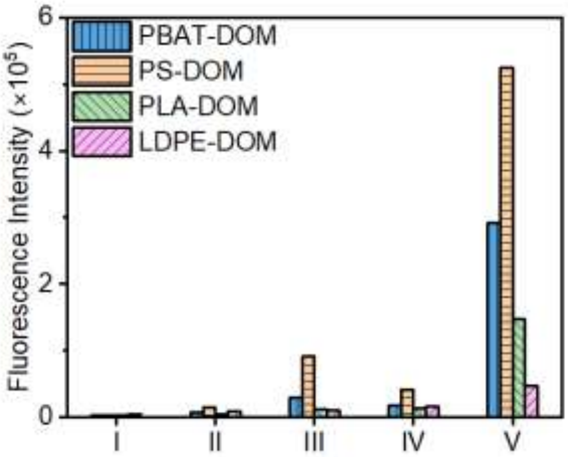

Figure S11. Comparison of fluorescence intensities across different regions for PS-DOM (a), PBAT-DOM (b), PLA-DOM (c), and LDPE-DOM (d).

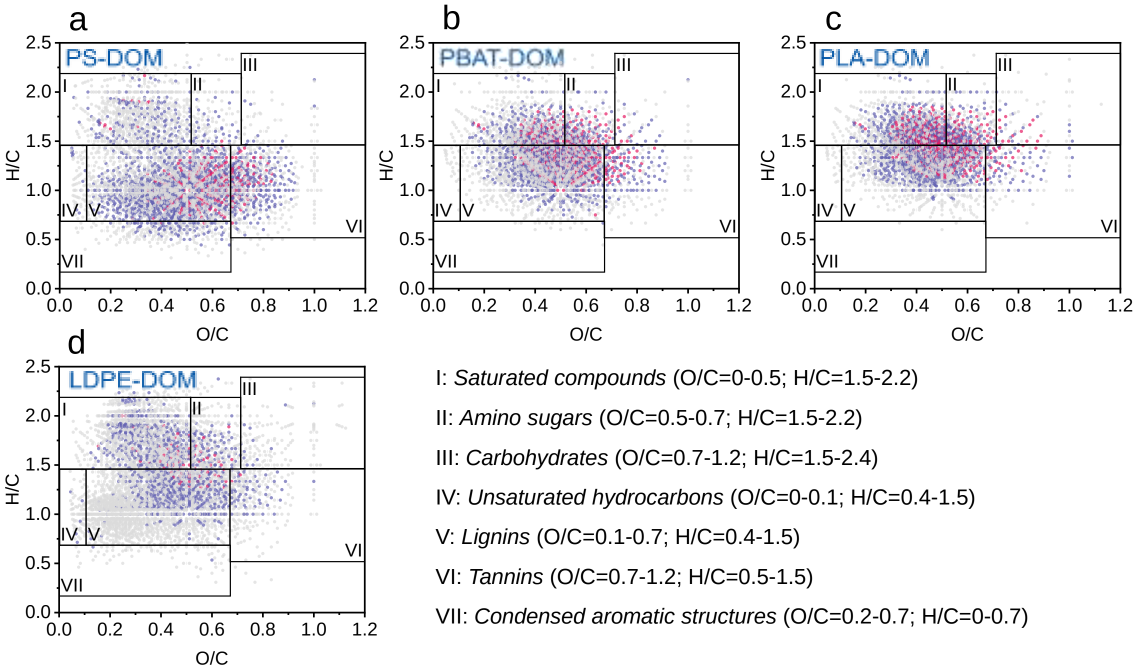

Figure S12. Van Krevelen diagrams depicting the molecular distribution and intensity characterization of PS-DOM (a), PBAT-DOM (b), PLA-DOM (c), and LDPE-DOM (d). Distinct color bars are applied in the diagrams to represent the three intensity ranks: Rank 1 (red) indicates intensity $> 1 \times 10^8$, Rank 2 (purple) indicates intensity between 1×10^7 and 1×10^8, and Rank 3 (gray) indicates intensity $< 1 \times 10^7$.

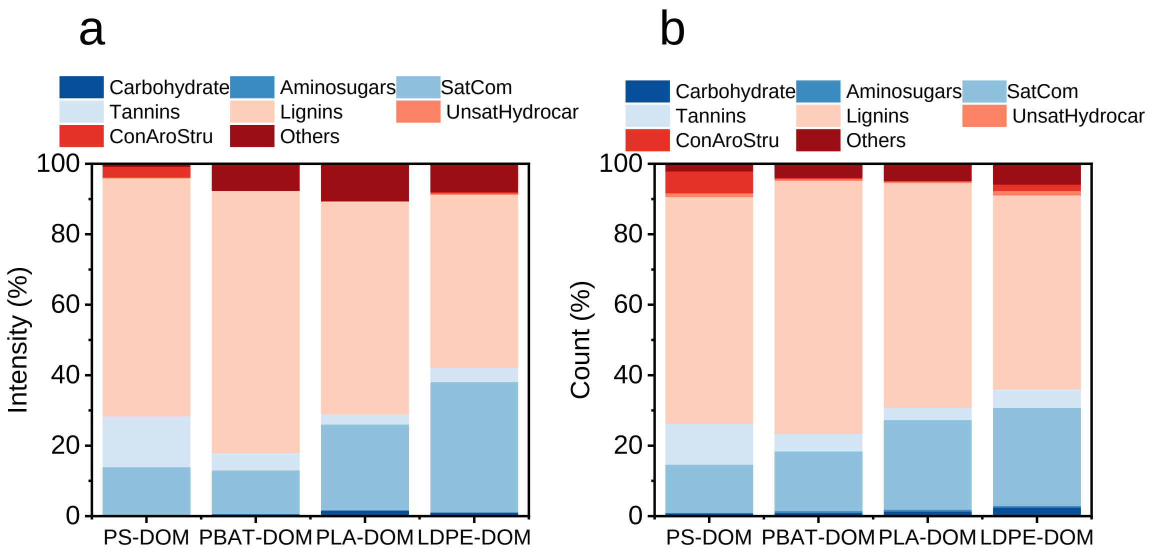

Figure S13. (a) Percentage intensity of different components in MP-DOM. (b) Percentage distribution of counts component in MP-DOM.

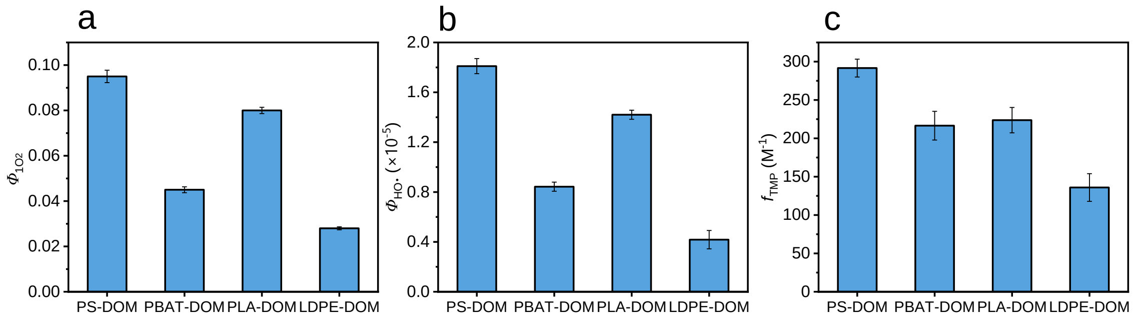

Figure S14. $\Phi_{^1O_2}$ (a), $\Phi_{HO\bullet}$ (b), and f_{TMP} values (c) of MP-DOM solution under simulated sunlight.

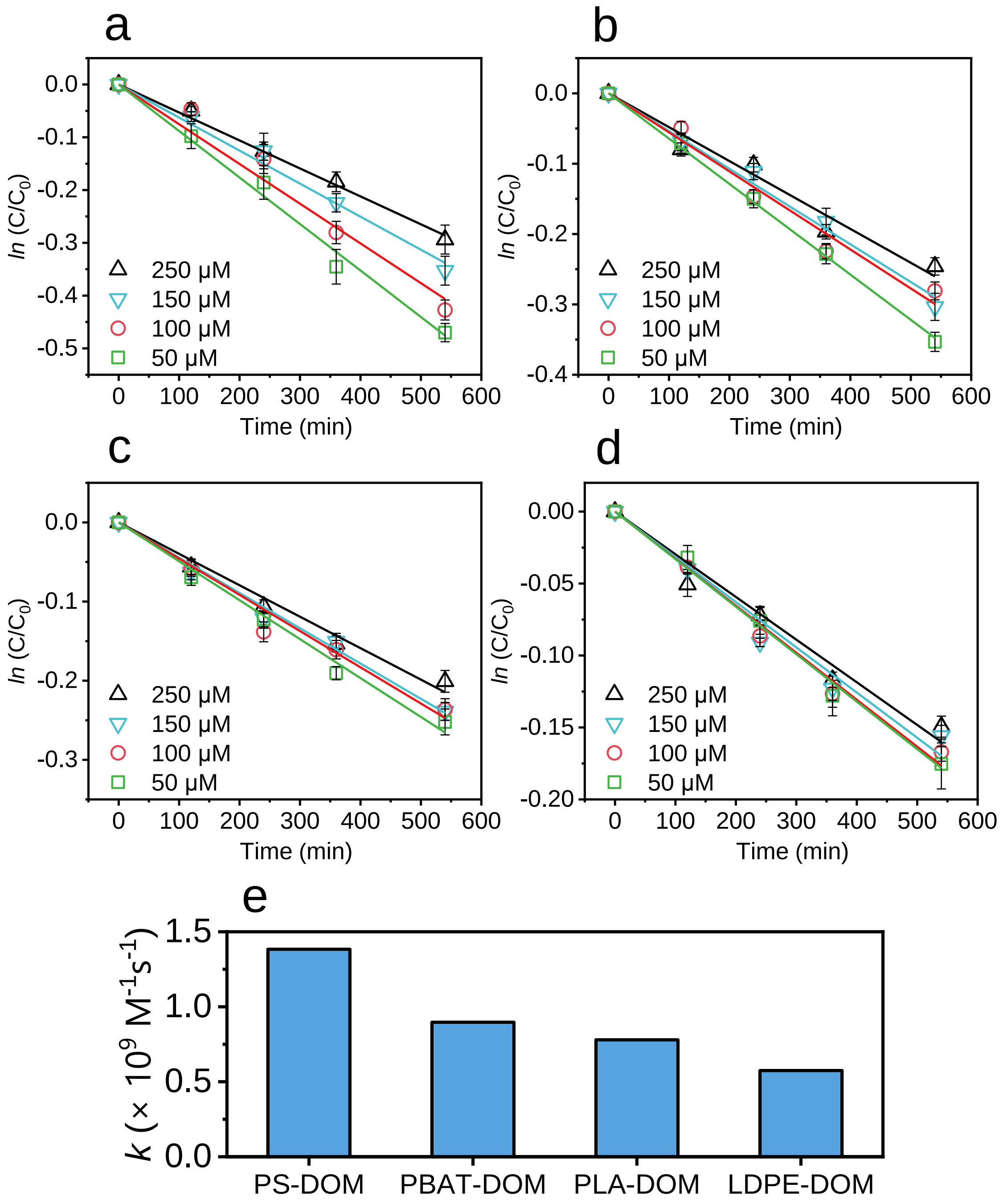

Figure S15. Photodegradation kinetics of various concentrations of TMP in PS-DOM (a), PBAT-DOM (B), PLA-DOM (C), and LDPE-DOM (D) irradiation systems. (e) Second-order kinetics of TMP with MP-DOM. Conditions: $[\text{MP-DOM}]_0 = 5.0\ \text{mg}_\text{C}\ \text{L}^{-1}$, pH 8.0.

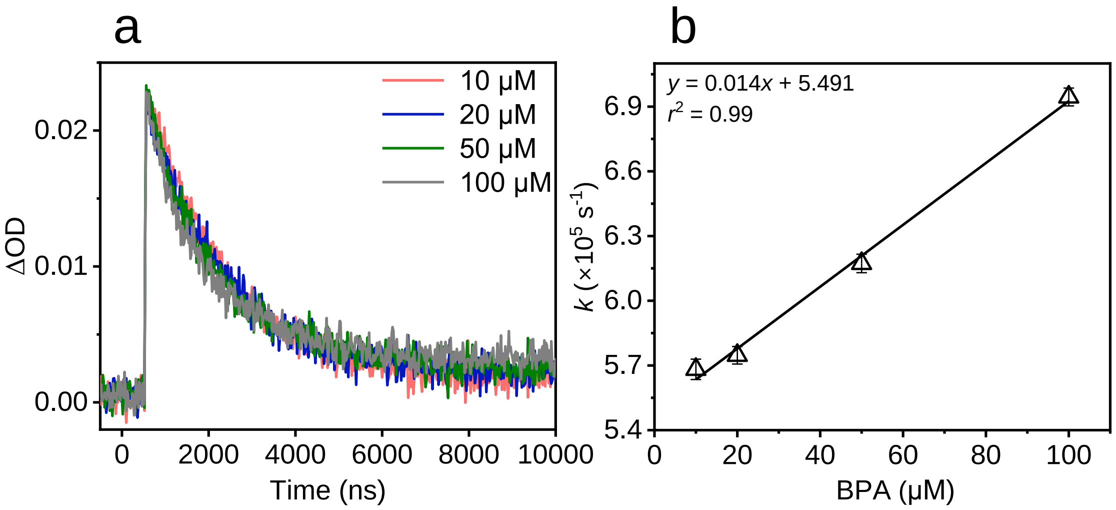

Figure S16. (a) Transient decay kinetics of ^{3}CBBP* at 550 nm with different concentrations of BPA. (b) Plot of the first-order decay rate constant k_{obs} *vs.* BPA concentration.

References

(1) Chen, W.; Westerhoff, P.; Leenheer, J. A.; Booksh, K. Fluorescence excitation−emission matrix regional integration to quantify spectra for dissolved organic matter. *Environ. Sci. Technol.* **2003**, *37*, (24), 5701–5710.

(2) McCabe, A. J.; Arnold, W. A. Reactivity of triplet excited states of dissolved natural organic matter in stormflow from mixed-use watersheds. *Environ. Sci. Technol.* **2017**, *51*, (17), 9718–9728.

(3) Golanoski, K. S.; Fang, S.; Del Vecchio, R.; Blough, N. V. Investigating the mechanism of phenol photooxidation by humic substances. *Environ. Sci. Technol.* **2012**, *46*, (7), 3912−3920.

(4) Wenk, J.; Canonica, S. Phenolic antioxidants inhibit the triplet-induced transformation of anilines and sulfonamide antibiotics in aqueous solution. *Environ. Sci. Technol.* **2012**, *46*, (10), 5455–5462.

(5) Wenk, J.; Eustis, S. N.; McNeill, K.; Canonica, S. Quenching of excited triplet states by dissolved natural organic matter. *Environ. Sci. Technol.* **2013**, *47*, (22), 12802–12810.

(6) Peng, J.; Pan, Y.; Zhou, Y.; Lei, X.; Guo, Y.; Lei, Y.; Kong, Q.; Cheng, S.; Yang, X. Mechanistic aspects of photodegradation of deoxynucleosides induced by triplet state of effluent organic matter. *Environ. Sci. Technol.* **2024**, *58*, (10), 4751–4760.

(7) Zhou, Y.; Zhou, Y.; Yao, L.; Luo, X.; Kong, Q.; Leung, K. S.-Y.; Yang, X. Insights into DOM-enhanced periodate degradation of emerging contaminants: The organic fenton-like reactions. *Environ. Sci. Technol.* **2025**, *59*, (40), 21682–21690.

(8) Lei, Y.; Yu, Y.; Lei, X.; Liang, X.; Cheng, S.; Ouyang, G.; Yang, X. Assessing the use of probes and quenchers for understanding the reactive species in advanced oxidation processes. *Environ. Sci. Technol.* **2023**, *57*, (13), 5433−5444.

(9) Ren, W.; Cheng, C.; Shao, P.; Luo, X.; Zhang, H.; Wang, S.; Duan, X. Origins of electron-transfer regime in persulfate-based nonradical oxidation processes. *Environ. Sci. Technol.* **2022**, *56*, (1), 78−97.

(10) Jiang, H.; Zhao, M.; Hong, W.; Song, W.; Yan, S. Mechanistic and kinetic consideration of the photochemically generated oxidative organic radicals in dissolved black carbon solutions under simulated solar irradiation. *Environ. Sci. Technol.* **2023**, *58*, (1), 760–770.

(11) Walpen, N.; Schroth, M. H.; Sander, M. Quantification of phenolic antioxidant moieties in dissolved organic matter by flow-injection analysis with electrochemical detection. *Environ. Sci. Technol.* **2016**, *50*, (12), 6423–6432.
